# Mesenchymal stem cells lose the senescent phenotype under 3D cultivation

**DOI:** 10.1186/s13287-023-03599-8

**Published:** 2023-12-18

**Authors:** O. Krasnova, A. Kovaleva, A. Saveleva, K. Kulakova, O. Bystrova, M. Martynova, A. Domnina, J. Sopova, I. Neganova

**Affiliations:** Institute of Cytology, Russian Academy of Sciences, Saint-Petersburg, Russia

**Keywords:** Three-dimensional cell culture, Mesenchymal stem cells, Senescence, Spheroids, Actin cytoskeleton

## Abstract

**Background:**

Three-dimensional (3D) cell culture is widely used in various fields of cell biology. In comparison to conventional two-dimensional (2D) cell culture, 3D cell culture facilitates a more accurate replication of the in vivo microenvironment, which is essential for obtaining more relevant results. The application of 3D cell culture techniques in regenerative medicine, particularly in mesenchymal stem cell (MSC)-based research, has been extensively studied. Many of these studies focus on the enhanced paracrine activity of MSCs cultured in 3D environments. However, few focus on the cellular processes that occur during 3D cultivation.

**Methods:**

In this work, we studied the changes occurring within 3D-cultured MSCs (3D-MSCs). Specifically, we examined the expression of numerous senescent-associated markers, the actin cytoskeleton structure, the architecture of the Golgi apparatus and the localization of mTOR, one of the main positive regulators of replicative senescence. In addition, we assessed whether the selective elimination of senescent cells occurs upon 3D culturing by using cell sorting based on autofluorescence.

**Results:**

Our findings indicate that 3D-MSCs were able to lose replicative senescence markers under 3D cell culture conditions. We observed changes in actin cytoskeleton structure, Golgi apparatus architecture and revealed that 3D cultivation leads to the nuclear localization of mTOR, resulting in a decrease in its active cytoplasmic form. Additionally, our findings provide evidence that 3D cell culture promotes the phenotypic reversion of senescent cell phenotype rather than their removal from the bulk population.

**Conclusion:**

These novel insights into the biology of 3D-MSCs can be applied to research in regenerative medicine to overcome replicative senescence and MSC heterogeneity as they often pose significant concerns regarding safety and effectiveness for therapeutic purposes.

**Supplementary Information:**

The online version contains supplementary material available at 10.1186/s13287-023-03599-8.

## Background

Mesenchymal stem cells (MSCs) are widely recognized as the most promising and frequently utilized cell type in regenerative medicine [1]. At the very beginning, MSCs were obtained from bone marrow, but currently MSCs can be harvested from various sources such as umbilical cord, adipose tissue, and Wharton Jelly [2–4].

MSCs are a type of somatic cells that have a limited number of divisions due to the Hayflick limit [5]. Once these cells reach their limit, they undergo replicative senescence, which is characterized by a flat and enlarged morphology, granulated cytoplasm, high activity of senescence-associated β-galactosidase (SA-β-gal), and increased lysosomal mass [6]. These changes, along with altered gene and surface marker expression and apoptosis resistance, collectively represent the replicative senescence phenotype [7, 8].

To overcome this senescence, approaches towards rejuvenation (that is, regaining the indefinite life span) of MSCs were undertaken with various methods such as 3D culturing, MSCs reprogramming, senolytic treatment, and regulation of miRNA expression are being explored [9–13].

Moreover, long-term cultivation can lead to MSC heterogeneity in terms of cell morphology and physiology, which can affect the effectiveness of therapeutic applications that rely on MSC properties such as differentiation and immunomodulatory abilities. Therefore, heterogeneous MSCs pose safety and effectiveness concerns [14].The therapeutic potential of MSCs is attributed to their ability to self-renew, differentiate into multiple lineages, and exhibit trans-differentiation plasticity [15, 16]. Moreover, MSCs possess the ability to migrate into injured sites and exhibit immunosuppressive functions in vivo [17, 18]. These features of MSCs enable the application of MSC-based therapy for treatment of various acute and chronic ailments, such as type 2 diabetes, graft-versus-host disease, Crohn disease [19–21]. The use of MSCs for the therapeutic purposes demands their expansion in vitro. However, despite the ability of MSCs to divide readily, the obtaining of large number of clinically-graded MSCs represents a clear challenge at the present state of the affairs.

There are multiple approaches to obtain clinically relevant cell number, including conventional two-dimensional (2D) culturing, three-dimensional (3D) culturing, and their modifications. Among these options, 3D culturing is currently the most promising technique for expanding MSCs as it allows to recapitulate the in vivo MSC niche [22, 23]. Furthermore, 3D-MSCs have been shown to possess enhanced therapeutic properties, whereas 2D-MSCs tend to exhibit early senescence [10, 24]. This deterioration commonly leads to low cell yield and decline in MSCs therapeutic potential [25].

Drastic changes in cell morphology can be observed during 3D culturing [10, 24]. These cells undergo transition from a flat spindle-like shape to a rounded form with a marked reduction in cell volume. These changes are accompanied by the loss of tension in the F-actin cytoskeleton, which is essential for the formation of 3D-spheroids. Treatment with cytochalasin D, an actin polymerization inhibitor, disrupts 3D-spheroid aggregation, while the presence of nocodazole, a suppressor of microtubules, does not affect the ability of cells to aggregate [26]. Furthermore, MSCs in 3D-spheroids exhibit reduced levels of monomeric actin, while the amount of alpha-tubulin remains constant [27]. The actin cytoskeleton interacts with all inner cellular compartments and plays a crucial role in many cellular mechanisms [28, 29]. Therefore, it is essential to understand how these cytoskeletal changes influence intracellular processes in 3D-MSCs and exert the beneficial effect of the 3D culturing.

Golgi Apparatus (GA) is a complex and dynamic structure that plays a crucial role in the secretory pathway by regulating cargo processing, sorting, and trafficking [30]. GA is closely associated with the cytoskeleton as vesicular transport is mediated by microtubules and actin filaments, and the cytoskeleton regulates GA positioning [31]. Although the key functions of GA remain unchanged in eukaryotic cells, its structure can vary dramatically. For example, interphase and mitotic GA differ even within a single type of vertebrate cell [32]. During interphase, GA is ribbon-like and positioned in the perinuclear region, but it disassembles into separated stacks with further vesiculation and partitioning between daughter cells during mitosis [32]. Actin cytoskeleton perturbation can lead to alterations in GA ultrastructure, such as dilatation or fragmentation [31]. In addition to its strong connection with the cytoskeleton machinery, GA is also linked to the mammalian target of rapamycin (mTOR) pathway. It has been shown that the main components of mTOR complex 1 (mTORC1) are located on the Golgi Ribbon [33, 34]. Importantly, mTORC1 is a positive regulator of cell size and senescence in various types of cells, therefore linking GA to the overall cell function [35, 36].

We aimed to investigate the impact of 3D culturing on MSCs phenotype, with a particular focus on the MSC potential to osteogenic differentiation and wound healing potency. Furthermore, we explored the potential of 3D culturing to reverse senescence and decrease heterogeneity of human MSCs (hMSCs). To gain insight into the mechanisms behind this effect of 3D culture we examined the actin cytoskeleton, Golgi apparatus, and mTOR intracellular localization. Our findings provide valuable insights into the biology of 3D cultured MSCs.

## Materials and methods

### Cell culture

Adipose tissue-derived mesenchymal stem cells line 1,519,000,139 and line 1,515,000,031 were purchased from Pokrovsky Stem Cell Bank (St Petersburg, Russia) and were cultured in tissue culture flasks 75 (TPP, Switzerland) in a growth medium consisting of DMEM with low glucose (1 g/ml D-glucose) (Thermo Fisher Scientific, USA), 10% fetal bovine serum (Thermo Fisher Scientific, USA), 100 U/ml Penicillin–Streptomycin (Thermo Fisher Scientific, USA) at 37 °C and 5% CO_2_.

### 3D-spheroids formation and 3D-2D transition

Adherent MSCs (2D-MSCs) were cultured 14 and more passages (considered as late passage here and after); after they have reached 80% of confluence, they were washed with phosphate buffered saline (PBS) (Merck, USA) and dissociated with 0.25% trypsin (Thermo Fisher Scientific, USA)-EDTA solution (PanEco, Russia) for 4 min at 37 °C. MSCs were cultured in 3D culture (3D-MSCs) in hanging drops in 25 μl DMEM with 10% FBS (Thermo Fisher Scientific, USA), 7000 cells per drop and incubated for 72 h at 37 °C and 5% CO_2_. To obtain 3D-2D MSCs, spheroids were incubated with 0.25% trypsin-EDTA solution for 7 min while pipetting every 3–4 min to dissociate. 3D-2D MSCs were cultured in tissue culture flasks as described above. In order to inhibit autophagy chloroquine (Merck, Germany) (inhibitor of autophagy) was added to cell suspension before 3D spheroids formation in its final concentration 5 μM [37].

### Immunofluorescence

2D-MSCs were grown on coverslips until they have reached 70% of confluence, and then they were processed for immunofluorescence. 3D-spheroids after 48 h of culture were embedded in pitch tissue-tek (Sakura, Japan) and sliced by microtome (Bright, UK) and slices (10 μm) were placed on a slide. To proceed further, cells on coverslips and slices on slides were washed with PBS once to be fixed with 2% of polyformaldehyde (PFA) during 15 min. After permeabilization with 0.025% of Triton X-100 in 30 min, the samples were blocked with 3% of normal goat serum (Abcam, UK) to avoid non-specific antibodies binding. Then the samples were incubated with the desired primary antibodies at 4 °C overnight. Next, the samples were gently washed with PBS and stained with the fluorophore-conjugated secondary antibodies. Nuclei were stained with 4, 6‐diamidino‐2‐phenylindole (DAPI) (Merck, USA). Images were collected using a confocal laser scanning microscope Olympus FV 3000 (Olympus, Japan). As primary antibodies, we have used: Anti-vinculin (Santa Cruz Biotechnology. USA, sc-73614), anti-β-catenin (Abcam, UK, ab6302), anti-p230 (BD Biosciences, USA, 611,280), anti-mTOR (Cell signalling technology, 2983), anti-p62 (BD Biosceinces, USA, 610,832) and anti-rabbit Alexa488 (Thermo Fisher Scientific, USA, A-11008), anti-mouse Alexa488 (Thermo Fisher Scientific, USA, A-11001) and anti-rabbit Alexa594 (Thermo Fisher Scientific, USA, A-11012) were used as secondary antibodies. F-actin was stained with rhodamine phalloidin (Thermo Fisher Scientific, USA, R415).

### Immunophenotyping

Immunophenotyping (CD markers expression) of 2D-MSCs, 3D-MSCs (48 h), and 3D-2D-MSCs was performed by flow cytometer CytoFlex (Beckman Coulter, USA). Single cell suspension of MSCs was obtained with 0.25% trypsin-EDTA solution to make 10^6^ cells/ml in PBS containing 0.5% bovine serum albumin (BSA) (Dia-M, Russia) and 0.1% NaN_3_. Cell suspensions were incubated with desired antibodies for 45 min at 4 °C. After incubation the suspensions were diluted 10 times in PBS. Four thousand events were analysed. FITC-conjugated antibodies to CD31 (BD Biosciences, USA), CD146 (Beckman Colter, USA), and phycoerythrin (PE)-conjugated antibodies to CD73 (BD Biosciences, USA), CD105 (BD Biosciences, USA), CD90 (BD Biosciences, USA), CD34 (BD Biosciences, USA), and HLA-DR (BD Biosciences, USA) were applied.

### Growth curve

To assess growth rate of 2D-MSCs (6p and 16p) and 3D-2D MSCs (1p), cells were seeded at 2*10^4^ cells/well in twelve-well plates. Every 24 h, the cell number of 3 wells were counted until 96 h. The growth curve based on the data was drawn and doubling time was counted using GraphPad Prism 9 (GraphPad Software Inc., USA).

### Senescence-associated β-galactosidase assay flow cytometry

To assess the activity of senescence-associated β-galactosidase (SA-β-gal) by flow cytometry CellEvent Senescence Green Flow Cytometry assay kit (Thermo Fisher Scientific, USA) was used. 2D-MSCs grown on 12 wells plate (TPP, Switzerland) for up to 80% of confluence were suspended with 0.25% trypsin-EDTA solution and fixed with 2% PFA for 10 min at 25 °C. Then MSCs were washed three times with PBS containing 1% BSA. After washing, cell pellet was suspended in working solution, containing 1 part of CellEvent Senescence Green Probe and 1000 parts of CellEvent Senescence Buffer to make cell suspension with 10^4^ cells in 30 μl of working solution. MSCs were incubated for 1 h at 37 °C without CO_2_. Stained cells were washed with PBS containing 1% BSA. SA- β-gal activity was assessed by flow cytometer CytoFlex (Beckman Coulter, USA).

### Senescence-associated-β-galactosidase assay

SA-β-gal activity also was evaluated with senescence galactosidase staining kit (Cell Signaling Technologies, USA). 2D-MSCs grown on 6 wells plate (TPP) for up to 70–80% of confluence were washed with PBS and fixed with 10% of fixation solution diluted in deionized water. After 20 min of incubation at 25 °C MSCs were stained with 20 mg/ml X-gal diluted in the staining buffer for 24 h at 37 °C without CO_2_. SA- β-gal activity was detected by EVOS Cell Imaging system (Thermo Fisher Scientific, USA).

### Lysosomal compartments staining

Lysosomes were stained with LysoTracker Red DND-99 (Thermo Fisher Scientific). MSCs grown on 6 wells plate (TPP) for up to 70–80% of confluence. Growth media then was removed and working solution was added: 100 nM of LysoTracker Red DND-99 diluted in the growth media. Cells were incubated for 1 h at 37 °C with 5% CO_2_ and then working solution was removed with fresh media. Stained cells were observed using EVOS Cell Imaging system (Thermo Fisher Scientific) fitted with 590 nm laser.

### MSCs differentiation

Osteogenic differentiation: MSCs grown on 24 wells plate (TPP, Switzerland) for up to 80% of confluence. Then growth media was replaced with StemPro™ Osteogenesis Differentiation media (Gibco, Thermo Fisher Scientific, USA). MSCs were refeed every 3–4 days. After 21 days of osteogenic differentiation induction MSCs were stained with Alizarin Red S to stain calcium deposits, marker of mature osteoblasts [38].

Chondrogenic differentiation: MSCs grown on 24 wells plate (TPP, Switzerland) for up to 80% of confluence. Then growth media was replaced with MSCgo™ Chondrogenic Differentiation Medium (Sartorius, Germany). MSCs were refeed every 3–4 days. After 21 days of chondrogenic differentiation induction MSCs were stained with Alcian Blue to stain acidic polysaccharides [39].

Adipogenic differentiation: MSCs grown on 24 wells plate (TPP, Switzerland) for up to 80% of confluence. Then growth media was replaced with MesenCult™ Adipogenic Differentiation Kit (Human) (STEMCELL Technologies Inc., Canada). MSCs were refeed every 3–4 days. After 21 days of adipogenic differentiation induction MSCs were stained with Oil Red O to lipid drops [40].

### Chromosome banding analysis

Chromosome banding analysis (G-banding) was performed for metaphase chromosomes of MSCs at early passage and after 3D-2D transition as described [41].

### Electron microscopy

2D-, 3D- (48 h), 3D-2D-MSCs were fixed with 2,5% glutaraldehyde in 0,1 M cacodylate buffer for 1 h. Then cells were postfixed in 1% OsO4 for 1 h. After three PBS washes, the cells were dehydrated through a graded series of 30–100% ethanol with further Epon-Araldite embedding. Ultrathin slices were cut by ultramicrotome (LKB, Sweden) and contrasted with 2% uranyl acetate in 50% methanol for 15 min, followed by 1% lead citrate for 10 min. Slices were viewed with Zeiss Libra 120 (Carl Zeiss, Germany) electron microscope at 80 kV. Ultrathin slices were treated with 3% hydrogen peroxide for 20 min and incubated with primary monoclonal antibody mTOR (Cell Signaling Technology, 2983) overnight in a moist chamber at 4 °C. After rinsing in PBS containing 0.1% fish gelatin and 0.05% Tween-20, the sections were incubated with secondary anti-rabbit antibodies conjugated to 10 nm colloidal gold particles (Sigma, Canada). The sections were contrasted with 2% uranyl acetate in 50% methanol for 15 min, followed by 1% lead citrate for 10 min. Slices were viewed with Zeiss Libra 120 (Carl Zeiss, Germany) electron microscope at 80 kV.

### Wound healing assay

2D-MSCs (at passage 16) and 3D-2D MSCs (at passage 1) were grown on µ-Dish 35 mm with removable silicone gasket with two 70 μm wells (Ibidi, Germany) for up 100% of confluence. Silicone insert was then removed to leave a defined gap (of 500 μm). Then each dish was filled with medium and placed on a microscope stage equipped with a climate-controlled chamber CellVoyager CQ1 (Yokogawa, Japan). Wound area was examined in real-time for the next 30 h, by taking images every 30 min.

### Real-time PCR analysis

To analyze gene expression, total RNA was isolated with Aurum™ Total RNA Mini Kit (Bio-Rad Laboratories, USA) according to the manufacturer’s instructions. RNA was quantified in the NanoDrop ND-1000 Spectrophotometer (Thermo Fisher Scientific, USA). cDNA was obtained by reverse transcription of RNA using the RevertAid H Minus First Strand cDNA Synthesis Kit (Thermo Fisher Scientific, USA) according to the manufacturer’s instructions. For qRT-PCR cDNA was amplified with specific primers, using qPCRmix-HS SYBR (Evrogen, Russia) in the Bio-Rad CFX Opus-96 real time system (Bio-Rad Laboratories, USA), according to the kit’s enclosed protocol. Expression of target genes was normalized to GAPDH gene. Expression in MSC at early (6p) passage was applied as control. Normalization was calculated using the ΔΔCt method. All amplifications were performed in 3 technical replicas. All experiments were performed as three biological repeats.

### Western immunoblotting

Total cell lysates were prepared by incubation of cells in RIPA lysis buffer containing protease inhibitors (Thermo Fisher Scientific, USA). Western blotting was performed using standard protocol. As primary antibodies we used: anti-β-catenin (Abcam, UK, ab6302), anti-vinculin (Santa Cruz Biotechnologies, sc-73614), anti-mTOR (Cell Signaling Technology, 2983), p-mTOR(S2448) (Cell Signalling Technology, USA, 2971S), anti-beta actin (Abcam, UK, ab8227), anti-p62 (BD Biosceinces, USA, 610,832). As a secondary antibody we used: Goat Anti-Rabbit IgG (H + L)-HRP Conjugate (Bio-Rad Laboratories, #1,706,515) and Goat Anti-Mouse IgG (H + L)-HRP) (Abcam, UK, ab205719). As a loading control we used GAPDH (Abcam, UK, ab8245). All experiments were performed as three biological repeats. Quantifications of western blot data were performed with ImageJ (Rasband, W.S., ImageJ, U. S. National Institutes of Health, Bethesda, Maryland, USA). Protein level in MSC at early (6p) passage was applied as control.

### Cell sorting

MSCs sorting based on autofluorescence was performed as described [42] using S3e Cell Sorter (Bio-Rad Laboratories, USA). Briefly, the cohort of MSCs at late [16] passage was divided into to two subpopulations according to cells size (FSC) and autofluorescence (inner granularity—SSC). MSCs with high FSC and SSC rates corresponded to senescent MSCs, while low FSC and SSC rates correlated with ‘early passage’ phenotype MSCs. MSCs with ‘senescent phenotype’ was stained with 500 nM CellTracker Deep Red dye (Thermo Fisher Scientific, USA) for 30 min at 37 °C.

### Statistical analysis

All experiments were performed as three biological repeats. The data are presented as the mean ± standard deviation (SD). Comparison of means in the statistical analysis was performed applying one-way ANOVA. Data were analysed with GraphPad Prism 9 (GraphPad Software Inc., USA). Differences were considered significant at *p* < 0,05.

## Results

### MSCs lost senescence markers under 3D culturing

**Fig. 1:**
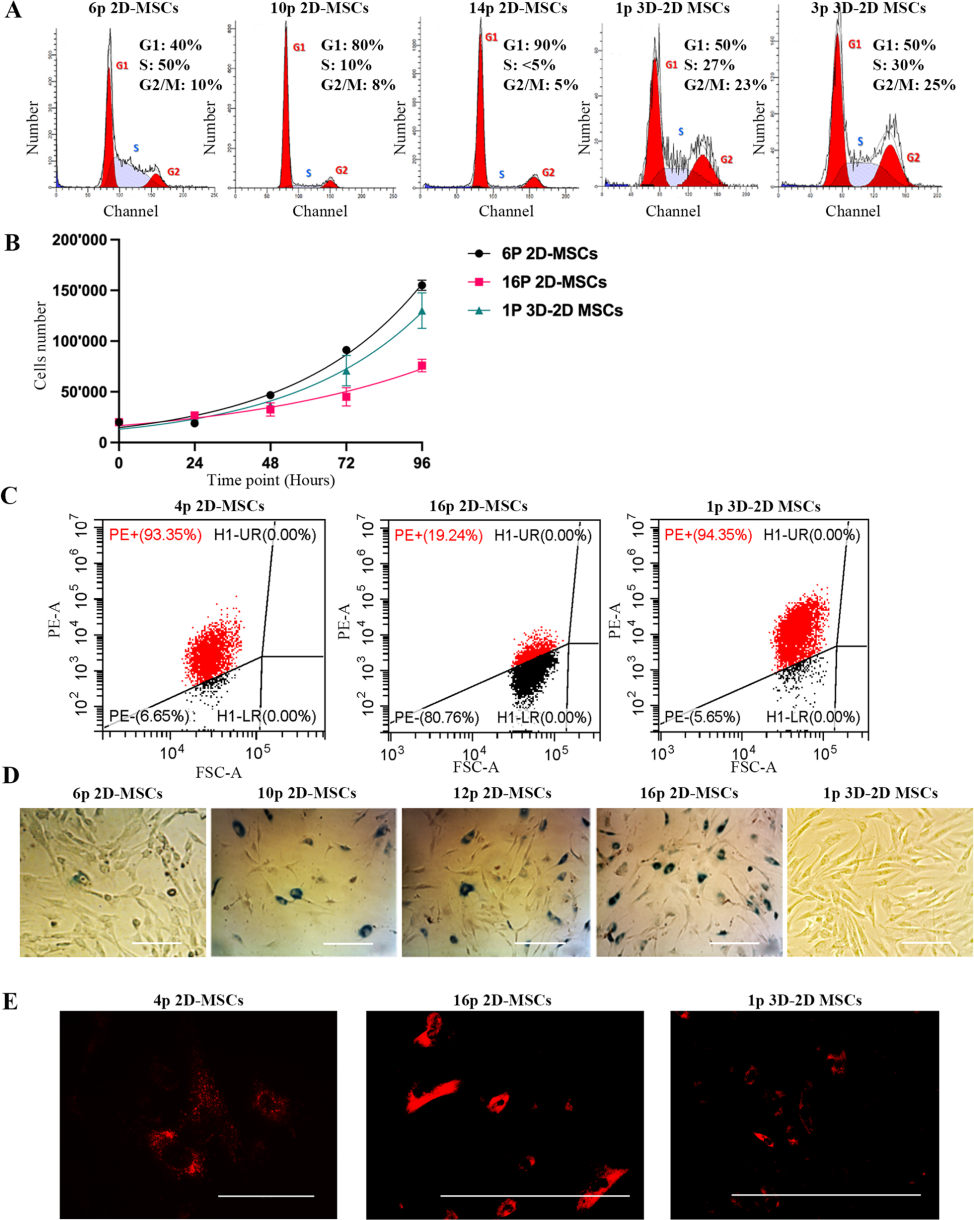
MSCs under 3D cell culture lost replicative senescence markers. (**A**): Cell cycle analysis of 2D-MSCs (6p, 10p, 14) and 3D-2D MSCs (1p and 3p). (**B**): The growth curve of 6p 2D-MSCs, 16p 2D-MSCs and 1p 3D-2D MSCs. Time point 24 h. (**C**): Flow cytometry analysis of CD146 surface expression in 2D-MSCs (4p and 16p) and 3D-2D MSCs (1p). (**D**): Analysis of senescence-associated (SA)-β-galactosidase activity in 2D-MSCs (6p, 10p, 12p, 16p) and 3D-2D MSCs (1p). Scale bar 50 μm. (**E**): Analysis of lysosomal activity in 2D-MSCs (4p and 16p) and 3D-2D MSCs (1p). Scale bar 100 μm

Our study revealed that prolonged 2D culturing of MSCs resulted in a higher proportion of cells in the G1/G0 phase of the cell cycle, indicating a state of quiescence. Specifically, after 4–7 passages, 50% of 2D-MSCs were in the DNA synthesis phase (S-phase) while 40% were in the G1/G0 phase (Fig. 1A). As the cells were cultured for longer, the percentage of cells in quiescence increased while the percentage of cells in DNA synthesis phase decreased. For instance, after 10 passages, only 15% of 2D-MSCs were in S-phase while 80% were in G1/G0. This trend continued, and after 14–16 passages over 90% of 2D-MSCs were in G1/G0 and less than 5% were in S-phase. However, when 2D-MSCs after 16 passages were cultured in 3D spheroids for three days and then transitioned back to 2D (3D-2D MSCs), there was a notable shift in cell cycle progression. The percentage of 3D-2D-MSCs in G1/G0 phase decreased from 90 to 50%, while the percentage in S-phase increased from less than 5–27% (Fig. 1A). This suggests that 3D culturing may have a positive effect on cell cycle progression and could potentially reverse senescence, as cell cycle arrest is a marker of replicative senescence [7] (Fig. 1A). However long-term 2D cultivation of 3D-2D MSCs inevitably leads to a reduction of MSCs in S-phase (Additional file 1: Fig.S1A). To support the obtained data, we examined the growth curve of 2D-MSCs after 6 and 16 passages, as well as 3D-2D MSCs after 1 passage (Fig. 1B). The growth curve analysis revealed that the mean population doubling time of 2D-MSCs (passage 6) was 28.11 h, which increased to 44.96 h for 2D-MSCs (passage 16). Interestingly, the mean population doubling time of 3D–2D MSCs decreased to 29.12 h, indicating an accelerated cell growth rate and enhanced cell proliferative ability after 3D culturing. Furthermore, we investigated in MSCs the expression of *CDKN1A* and *CDKN2A*, which code for the cell cycle inhibitors p21 and p16, respectively [43]. We observed an upregulation of *CDKN1A* in long-term cultured MSCs, while *CDKN2A* remained stable. Interestingly, when MSCs were cultured in a 3D environment, there was a significant increase in the expression level of both genes. However, upon transitioning back to a 2D culture, we observed a downregulation of these genes. Specifically, *CDKN1A* expression returned to levels similar to those seen in late passage MSCs, while *CDKN2A* expression dropped significantly below even the levels observed in early passage MSCs (Additional file 1: Fig. S1B). The data on cell cycle related genes aligns with evidence of increased proliferative ability in 3D-2D MSCs.

Cell cycle arrest is not the only marker of the replicative senescence. Low rate of surface expression of CD146, known as melanoma cell adhesion molecule is considered as another senescence marker [44]. Indeed, 93% of 2D-MSCs being cultured for 4–6 passages were CD146-positive, in agreement with the cell cycle data presented above (Fig. 1C). Further cultivation and passaging led to a decrease of the number of 2D-MSCs which were positive for CD146; 19% of 2D-MSCs after 16 passages had this marker on their surface. Given the effect of 3D culturing and 3D–2D transition on cell cycle progression, the surface expression of CD146 in 3D–2D MSCs was analyzed. It was found that 94% of 3D-2D MSCs possessed CD146 on their surface and it is fully consistent with the data of cell cycle analysis. Interestingly, only 19% of MSCs in 3D spheroids were positive for CD 146 (Fig. 1C), thus, the 3D–2D transition plays a key role in supporting the expression of CD146 on the surface (Additional file 1: Fig. S1C). As a control, the expression of another mesenchymal marker, CD90, was examined. Indeed, all three kinds of cells: 2D-MSCs (passage 4 and passage 16), 3D-MSCs, and 3D-2D MSCs exhibited the same level of CD90 on their surface (Additional file 1: Fig. S1D).

To further support the data on the negative impact of 3D cultivation on senescence, we analyzed senescence-associated (SA) β-galactosidase (SA-β-Gal) activity. 2D-MSCs after 4–6 passages showed low SA-β-Gal activity, with only 5% of cells at this cultivation stage being positive for this senescence marker (Fig. 1D). However, as the number of cells in G1/G0 increased, the number of cells positive for SA-β-Gal also raised. As expected, 25% of 2D-MSCs after 10 passages demonstrated elevated SA-β-Gal activity, and after 16 passages, more than 75% of 2D-MSCs were SA-β-Gal-positive. These cells with high levels of SA-β-Gal activity were then used for 3D spheroid formation. Following three days of 3D cultivation cells were then put back to 2D culture. In these 3D–2D MSCs the number of SA-β-Gal-positive cells was drastically reduced to about 1% of the population, as compared with 2D culture (Fig. 1D).

To validate our findings regarding the reversing effect of 3D culturing on senescence, the lysosomal activity was assessed as elevated lysosomal mass is a marker of senescent cells [45]. Using LysoTracker Red DND-99, we stained 2D-MSCs to specifically detect acidic lysosomal compartments. Microphotographs revealed that 2D-MSCs after 6 passages demonstrated a moderate number of lysosomes (Fig. 1E). However, prolonged 2D cultivation for 16 passages resulted in a significant increase in lysosomal mass in 2D-MSCs, this is consistent with previous findings that show senescence occurring after long-term cultivation. More importantly, late passage 2D-MSCs after 3D culturing and 3D–2D transition (3D–2D MSCs) exhibited similar lysosomal activity to that observed in earlier 2D-MSCs after only 6 passages (Fig. 1E), further supporting the hypothesis of a reversing effect of 3D culturing on senescence.

Thus, our findings suggest that the use of 3D cultivation techniques can aid in the rejuvenation of MSCs in vitro. This could have significant implications for the field of regenerative medicine, as the senescence of MSCs has been identified as a major obstacle to their clinical application.

### Human MSCs retain their phenotype and enhance osteogenic differentiation and migration ability upon 3D culturing

**Fig. 2.**
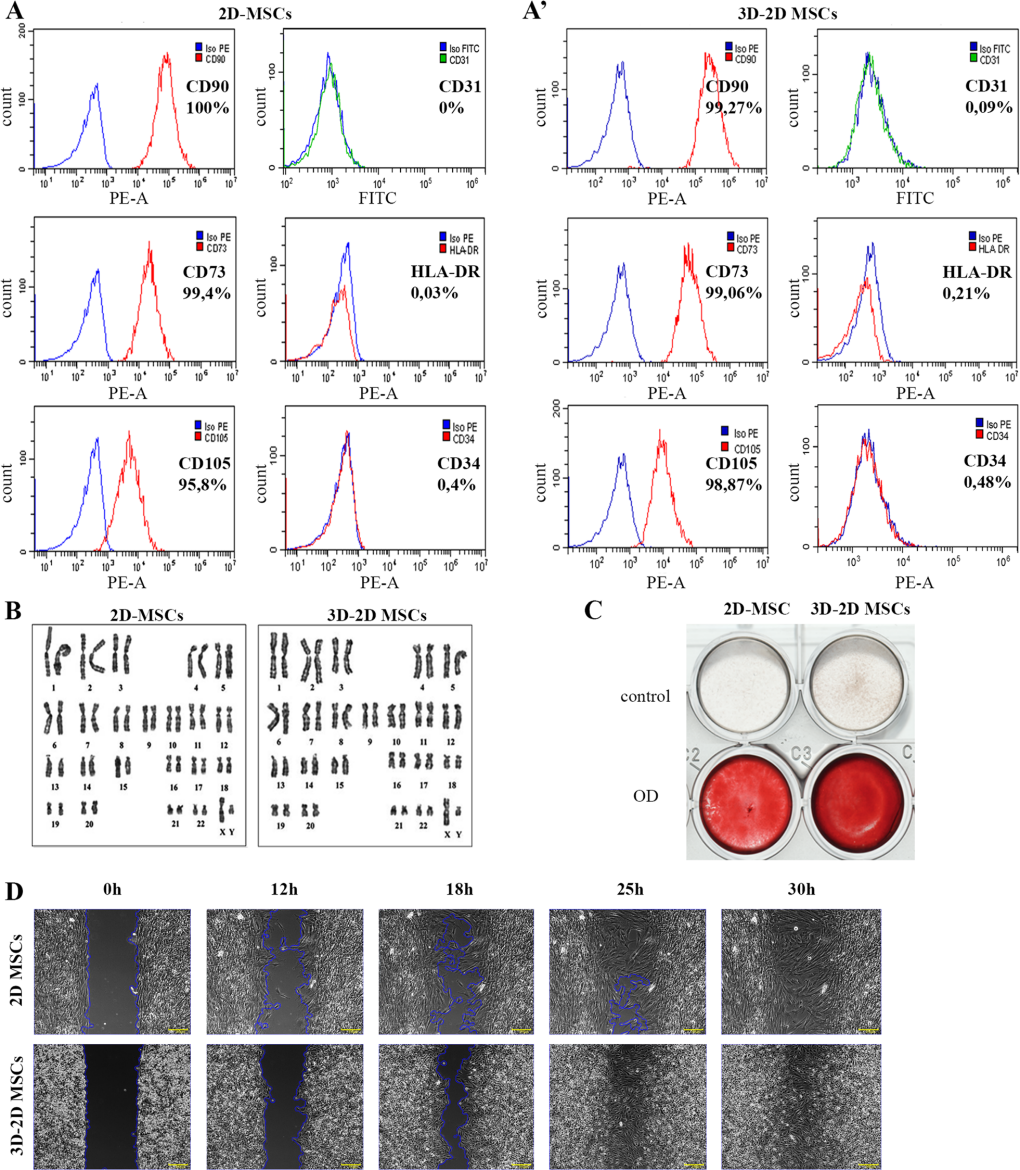
3D-2D MSCs compared to 2D-MSCs. (**A**): The immunophenotype of 2D-MSCs. (**A’**): The immunophenotype of 3D-2D MSCs. (**B**): Karyotyping analysis of 2D-MSCs and 3D-2D MSCs. (**C**): Analysis of osteogenic differentiation effectiveness (Alizarin Red staining for calcium deposits) of 2D-MSCs and 3D-2D MSCs. (D): Assessing of wound healing potency of 2D-MSCs and 3D-2D MSCs within 30 h. Scale bar 200 μm

To investigate the impact of 3D culturing on the phenotype of MSCs, we conducted an immunophenotypic analysis of cells cultured in 3D spheroids for 72 h followed by dissociation and 2D culturing (3D–2D-MSCs). The immunophenotype of 3D-2D MSCs was then compared to that of cells cultured only in 2D as a control (2D-MSCs). Our results revealed no significant difference in the surface expression of MSC markers: CD90(+), CD73(+) and CD105(+) between 2D-MSCs and 3D–2D MSCs. Furthermore, 3D–2D MSCs were also negative for the markers such as CD34(−), HLA-DR (−), and CD31(−) (Fig. 2A, A’). These findings suggest that 3D culturing does not alter the immunophenotype of MSCs.

To ensure the reliability of our findings, we performed karyotyping analysis on 3D-2D MSCs taken from spheroids. Our results indicate that the karyotype of these cells remained normal without any translocations, aneuploidy, deletions or additions (Fig. 2B), indicating the maintenance of genome stability.

Furthermore, the osteogenic differentiation assay showed that 3D–2D MSCs exhibited augmented osteogenic potential in comparison with 2D-MSCs (Fig. 2C), providing strong evidence for the retention and improving of their differentiation capacity. Also, 3D–2D MSCs retained the ability to differentiate into adipogenic and chondrogenic lineages (Additional file 2: Figs. S2A and S2B).

Active migration ability of MSCs is crucial for their use in regenerative medicine. To see whether the cultivation in 3D changes their migration ability we studied 3D–2D-MSCs. Our results demonstrated that 3D-2D-MSCs retained normal ability to migrate after the transition from 3 to 2D culture (Fig. 2D). Interestingly, we even observed a slight enhancement in migration ability in the cells that underwent the 3D–2D transition, as compared to those cultured solely in 2D. Specifically, 3D–2D MSCs were able to heal a wound in 22 h, while 2D-MSCs required 28 h to do so (Additional file 2: Fig. S2C). These results suggest that 3D culturing has no negative effect on the migration ability of 3D–2D MSCs and may even have a positive impact on this property.

### Cytoskeleton and adhesion proteins in MSCs under various culture conditions

**Fig. 3:**
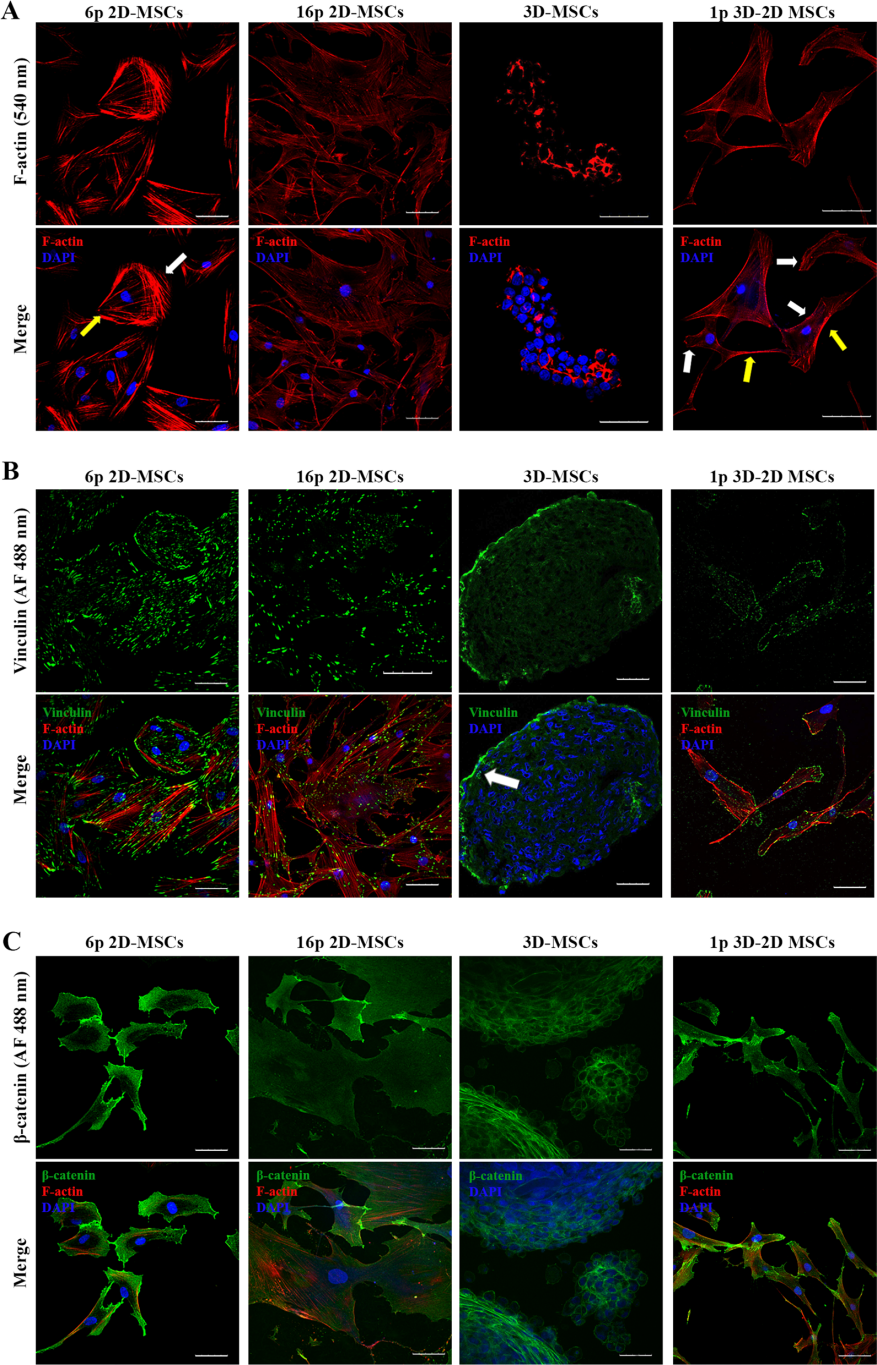
Immunofluorescent analysis of cytoskeletal and adhesion proteins in MSCs in various culture conditions (**A**): F-actin in 2D-MSCs (6p and 16p), 3D-MSCs (48 h) and 3D-2D MSCs (1p). White arrows point at leading edge, yellow arrows point at trailing edge. (**B**): Vinculin in 2D-MSCs (6p and 16p), 3D-MSCs (48 h) and 3D-2D MSCs (1p). White arrow points at the outer layer of spheroid. (**C**): β-catenin in 2D-MSCs (6p and 16p), 3D-MSCs (48 h) and 3D-2D MSCs (1p). Scale bar 50 μm. Abbreviations: DAPI—4′,6-diamidino-2-phenylindole

The fluorescence staining with rhodamine phalloidin revealed that after 6 passages 2D-MSCs exhibited a distinct distribution of actin filaments, with clear leading and trailing edges, which signifies that these cells are highly mobile (Fig. 3A). However, as the 2D-MSCs were cultured for 16 passages, the actin cytoskeleton structure underwent significant changes. Cells at later passage show signs of hypertrophy with abundant actin fibers which are not organized in a leading edge filopodia structure but rather distributed all along the cell body which is similar to what is observed in senescent cells [46]. Upon examining the actin structure in slices made of 3D-MSCs spheroids we observed condensation of filaments at the cell edges and at the cell–cell contacts; rhodamine phalloidin could stain these structures, thus actin during 3D culturing remains filamentous (Fig. 3A). Then, as 3D-MSCs were replated in dishes and underwent 3D–2D transition the actin cytoskeleton structure became again similar to that of 2D-MSCs after 6 passages, with well-ordered filaments and distinguishable leading and trailing edges (Fig. 3A).

Our fluorescence analysis of F-actin structure is consistent with qPCR analysis of *ACTB* expression (Additional file 3: Fig.S3A). Long-term 2D cultivation led to a slight upregulation of *ACTB* mRNA level as compared with the mRNA level in 2D-MSCs after 6 passages. Then, as cells were cultured in 3D spheroids, the *ACTB* expression became drastically downregulated and upon 3D–2D transition the gene expression was again upregulated (Additional file 3: Fig.S3A). Importantly, obtained data on gene expression and the fluorescent analysis correlate with western blot analysis; under 3D cell culturing a decline in β-Actin protein level is observed (Additional file 3: Fig. S3B).

Vinculin is a highly conserved focal adhesion protein which facilitates cell adhesion to the extracellular matrix via binding to actin and stimulation its polymerization [47]. We analyzed vinculin expression with immunofluorescence during 2D culture, 3D and 3D–2D cultures of MSCs. After 6 passages 2D-MSCs were characterized by pronounced focal adhesion plaques (Fig. 3B). Nevertheless, MSCs at each stage of 2D culturing possessed focal adhesion plaques. After analyzing the expression pattern of vinculin in 3D-MSCs, a drastic change was observed where only the outer layer of the 3D spheroid showed positive vinculin staining (Fig. 3B). We suggest that this change is because 3D-MSCs were no longer adherent to the substrate, but rather formed cell–cell contacts. This result correlates with the reduction of the F-actin structure in the 3D spheroids, as vinculin is involved in maintaining the tension of filamentous structure of actin [48].

Importantly, as 3D-MSCs underwent 3D–2D transition, the focal adhesions were restored at the leading edges of the cells. However, vinculin expression pattern was less pronounced as those in 2D-MSCs after 6 passages (Fig. 3B).

The immunofluorescence analysis of vinculin expression pattern correlated with the data on *VCL* gene expression (Additional file 3: Fig. S3A). 2D-MSCs after 6 passages exhibited the higher mRNA level of *VCL* in comparison to its level in 2D-MSCs after 16 passages. The downward trend retained after 3D spheroids formation which is also consistent with the immunofluorescence analysis. After 3D–2D transition 3D–2D -MSCs demonstrated upregulated *VCL* and the level of mRNA was similar to that in 2D-MSCs after 6 passages which implies on reverse senescence effect of 3D culturing. Moreover, it correlates with enhanced migration ability of 3D–2D-MSCs as mentioned above (Fig. 2D).

However, it is important to mention that total amount of vinculin protein remains stable throughout all the culture conditions, as western blot data demonstrates (Additional file 3: Fig. S3B). Vinculin is a focal adhesion protein as well as it involves in cadherin-cadherin adhesion. Thus, as under 3D culture condition cell–cell interactions via cadherins are increased, vinculin is involved in that type of interactions this result is not contradictory [10, 47].

As another example of adhesion-related marker, we assessed the expression of β-catenin using immunofluorescence and qPCR analysis. β-catenin is a multifunctional protein that plays a crucial role in cell–cell adhesion by binding the cytoplasmic domains of cadherins to the actin cytoskeleton [49]. Our immunofluorescent analysis showed that 2D-MSCs after 6 passages exhibited a pronounced β-catenin distribution pattern, in particular at the leading edge of migrating cells, while long-term 2D cultivation was associated with a decrease in β-catenin expression (Fig. 3C). Similarly, to vinculin, the decline in β-catenin expression was particularly evident after 16 passages. Evidently, 3D-MSCs showed a much-increased β-catenin staining, most likely as a result of an increase in the number of cell–cell contacts in spheroids (Fig. 3C) [10]. After replating in 2D, 3D–2D-MSCs exhibited a β-catenin expression pattern similar to that observed in 2D-MSC cells after 6 passages (Fig. 3C).

After examining gene expression, we found that after 16 passages, 2D-MSCs demonstrated a downregulation of *CTNNB1* compared to 2D-MSCs after 6 passages (Additional file 3: Fig. S3A), which is consistent with the immunofluorescent analysis. When MSCs were cultured in 3D spheroids for three days, we observed a three-fold increase in *CTNNB1* mRNA levels. This finding suggests that 3D culturing promotes enhanced cell–cell interaction. After transitioning from 3 to 2D culture, we detected a drastic decline in *CTNNB1* expression, similar to the pattern observed in cells after 6 and 16 passages. It is worth to note that western blot analysis partially correlates with those of the immunofluorescent and gene expression analysis: while we observed upregulation of *CTNNB1* in 2D-MSCs after 6 passages, the protein level was lower than that in 2D-MSCs passaged 16 times. Moreover, western blot analysis revealed that β-catenin in 3D-MSCs decreased and it elevated after 3D–2D transition (Additional file 3: Fig. S3B). β-catenin is a protein involved in cell–cell interactions as well as it is component of canonical The Wnt/β-catenin pathway which is beyond the scope of this research [38].

In light of our findings, it can be inferred that both cell—extracellular matrix (ECM) and cell–cell contacts and associated cytoskeletal components are sensitive to culture conditions. It can be proposed that significant alterations during 3D culturing are potentially related to the transitioning from cell-ECM adhesion to cell–cell adhesion.

### Organization of Golgi apparatus in MSCs under various culture conditions

Among other functions, the actin cytoskeleton plays a crucial role in the positioning of cell organelles [50]. As previously mentioned, 3D-MSC cells become more reliant on cell–cell adhesion, rather than cell-ECM adhesions. In contrast to 2D conditions where cells adhere to a substrate and exhibit polarity, 3D spheroids lack adhesion to a substrate and therefore cells do not exhibit migration or cell polarity [51]. Given the interconnection between migration, cell polarity and the Golgi apparatus, we investigated its organization in 2D-MSCs and 3D-MSCs and 3D–2D-MSCs [52].

**Fig. 4:**
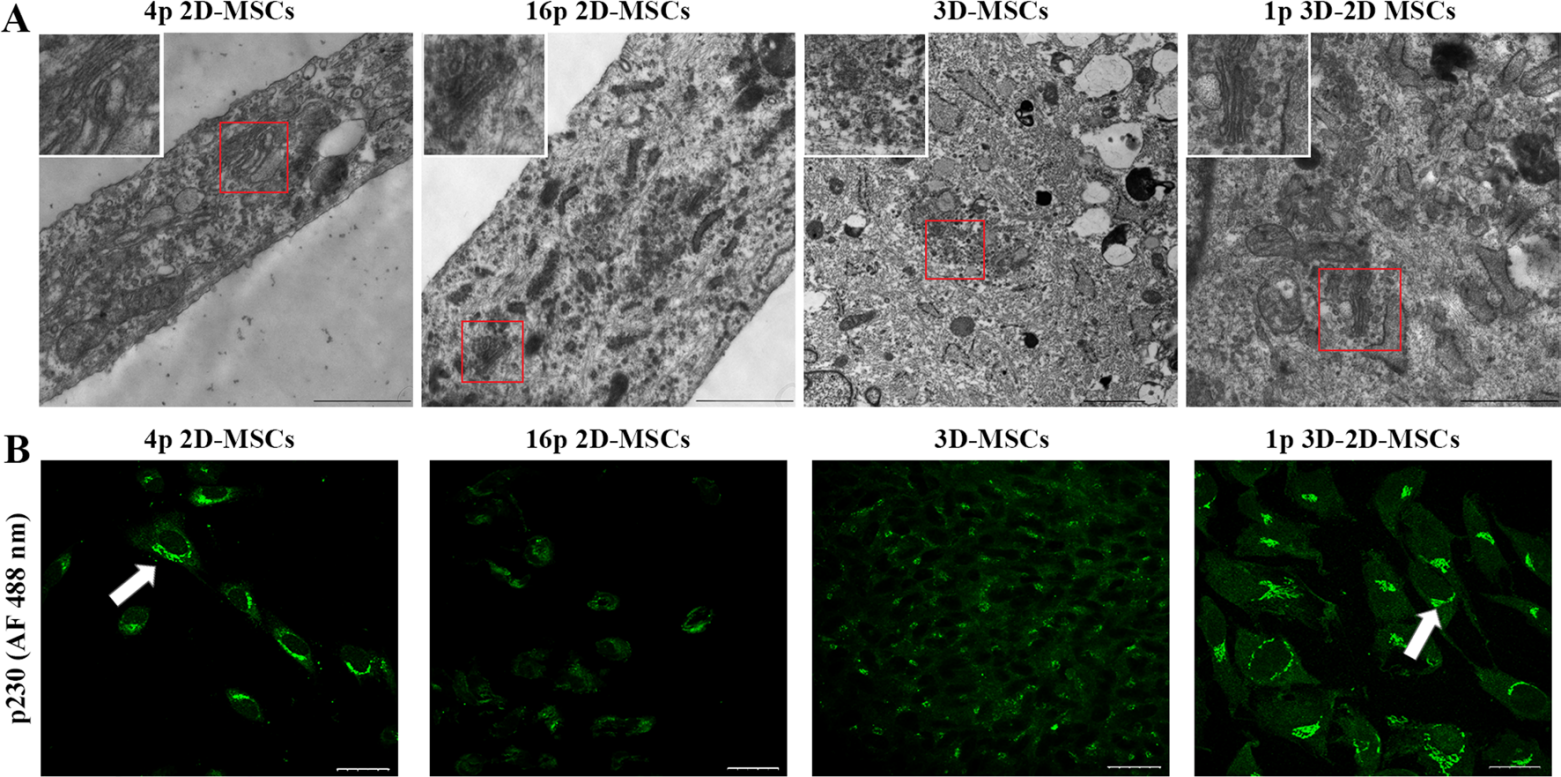
Golgi apparatus structure in MSCs in various culture conditions. (**A**): Electron microscopy observation of Golgi apparatus in 2D-MSCs (4p and 16p), 3D-MSCs (48 h) and 3D-2D MSCs (1p). Scale bar 1 μm. Red frames point at Golgi apparatus within cell, which is shown at higher magnification in the white inset. (**B**): Confocal immunofluorescence observation of p230 in 2D-MSCs (4p and 16p), 3D-MSCs (48 h) and 3D-2D MSCs (1p). Scale bar 50 μm

Electron microscopy (EM) revealed that MSCs possessed a well-organized Golgi apparatus composed of flattened membrane-enclosed sacs (cisternae) after 6 passages (Fig. 4A). However, prolonged 2D cultivation led to a dilatation of the Golgi apparatus structure, with a regular architecture being maintained but cisternae becoming enlarged. Upon transitioning to 3D cultivation, MSCs exhibited drastic changes in Golgi apparatus structure, with a fragmentation and a formation of Golgi mini-stacks. Only upon returning to 2D culture and adhering to the substrate did the Golgi apparatus regain its well-established structure, similar to that observed in 2D-MSCs after 6 passages (Fig. 4A).

We investigated the localization of the trans-Golgi network marker p230 by immunofluorescence analysis. Our findings showed that 2D-MSCs after 4 passages had compact perinuclear p230 localization, while those subjected to prolonged 2D cultivation exhibited a dispersed pattern of p230, indicating Golgi apparatus structural disturbances (Fig. 4B). In 3D spheroids, p230 localization was altered, with a dispersed cytoplasmic distribution and an absence of perinuclear localization (Fig. 4B). However, upon transitioning back to 2D culture and adhering to the substrate, the expression pattern of p230 was restored, with perinuclear and compact p230 distribution patterns.

### mTOR localizes in nucleus and nucleolus within 3D-MSCs

**Fig. 5:**
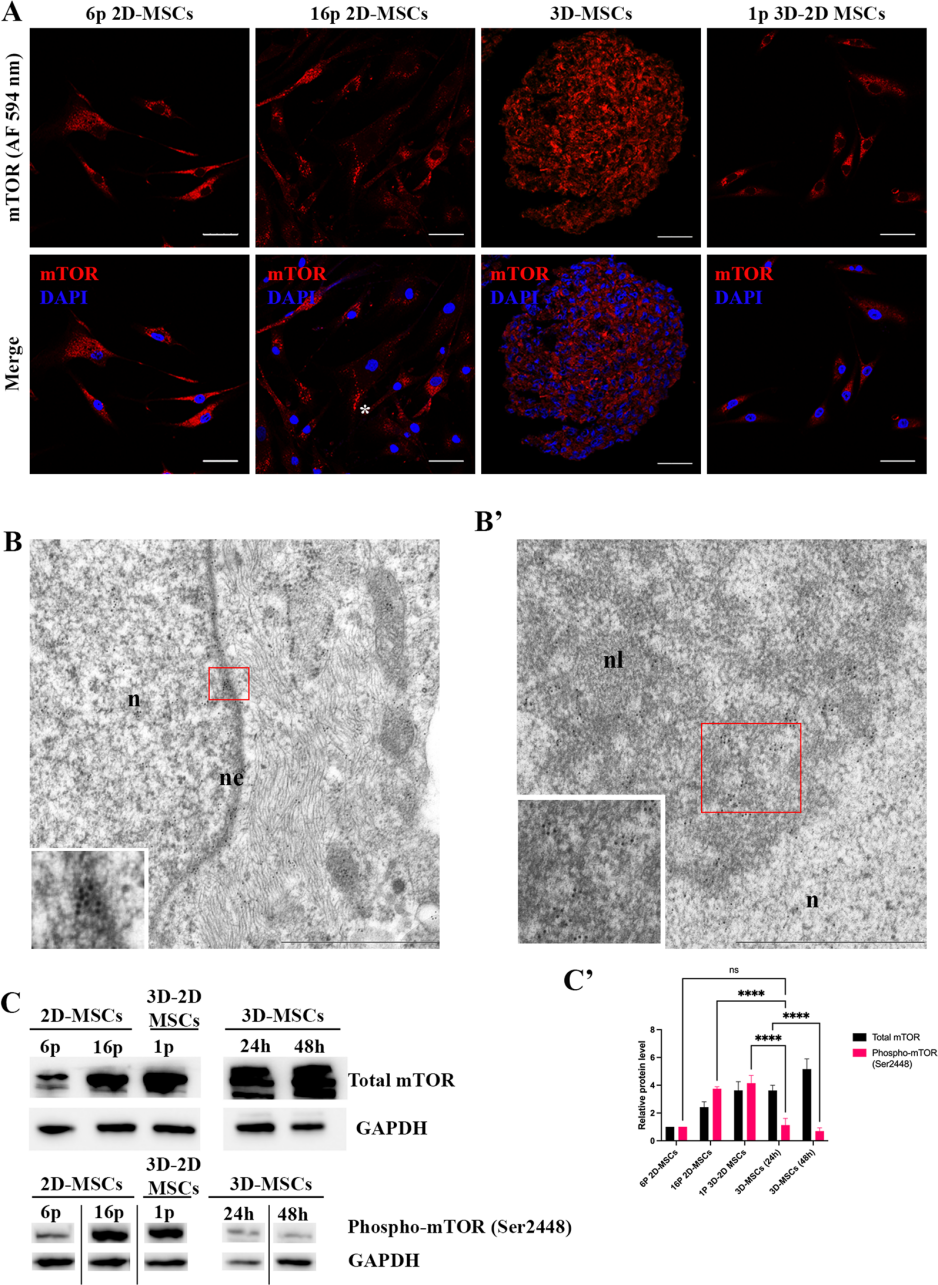
The localization of mTOR in MSCs in various culture conditions. (**A**): Confocal immunofluorescence observation of mTOR in in 2D-MSCs (4p and 16p), 3D-MSCs (48 h) and 3D-2D MSCs (1p). Asterix point at dispersed localization of mTOR in senescent cells. Scale bar 50 μm. (**B**, **B’**): Electron microscopy observation of mTOR in 3D-MSCs. Abbreviations: n stands for nucleus, nl—nucleolus, and ne—nuclear envelope. Scale bar 1 μm. Red frames point at mTOR immunogold labeling, which is shown at higher magnification in the white inset. (**C**, **C’**): Representative Western blot analyses of the mTOR, phospho-mTOR (Ser2448) in in 2D-MSCs (6p and 16p), 3D-MSCs (24 h and 48 h) and 3D–2D MSCs (1p). n = 3. Data are shown as mean ± SD, n = 3, with significance difference indicated with asterisks (ns—not significant, ****p < 0.0001). Abbreviation: GAPDH—glyceraldehyde-3-phosphate dehydrogenase. Full-length blots are represented in Additional file 6: Figure S1

The Golgi apparatus (GA) is a crucial organelle involved in the processing, modification, and packaging of proteins [30]. In addition to its primary functions, the GA also serves as a hub for various signaling molecules, including mTORC1 [34] (Additional file 4: Fig. S4). This protein complex is of particular interest to researchers as it plays a crucial role in regulating cellular size and senescence [35, 36]. We found in 2D-MSCs after 6 passages mTOR was mostly concentrated near the nucleus, but also dispersed in cytoplasm (Fig. 5A). However, after long-term 2D cultivation this dispersion increased—distribution pattern of mTOR scattered (Fig. 5A). These data correlated with senescence-associated Golgi fragmentation (Fig. 4B). Further investigation of 3D-MSCs revealed dispersed cytoplasmic localization (Fig. 5A). When 3D-MSCs were re-attached to substrate again, mTOR localization was observed similar to that in 2D-MSCs after 6 passages (Fig. 5A). In addition, we performed an immunoelectron microscopy to identify the localization of mTOR. Interesting insights revealed EM microphotographs: in 3D-MSCs mTOR was found in cytoplasm, nucleus and nucleoli, while adherent MSCs (2D cultured) had mTOR only in cytoplasm (Fig. 5B, B’).

In the cytoplasm under nutrient-sufficient conditions, mTOR is phosphorylated via PI3 kinase/Akt signaling pathway at Ser2448 [53]. Thus, we measured the amount of phospho-mTOR (Ser2448) and observed a drastic decline in 3D-MSCs as compared to 2D-MSCs and 3D-2D MSCs (Fig. 5C, 5’).

In MSCs, mTOR negatively regulated the expression of Transcription Factor EB (*TFEB)* gene [54]. Indeed, we observed upregulation of *TFEB,* in 3D-MSCs in comparison with 2D-MSCs and 3D-2D MSCs (Additional file 5: Fig. S5A). TFEB plays a crucial role in regulating various stages of autophagy, which can have both pro- and anti-senescence effects. Further analysis of gene expression revealed upregulation of *ATG5*, *ATG16*, *MAP1LC3A*, and *GABARAPL* genes involved in phagophore elongation (Additional file 5: Fig. S5A). Along with nuclear mTOR localization and upregulation of *TFEB*, EM microphotographs revealed increased number of double-layer membrane structures, autophagosomes, in 3D-MSCs (Additional file 5: Fig. S5B). Immunofluorescent and western blot analysis of the common autophagy marker p62 showed elevated levels in 3D-MSCs compared to 2D-MSCs, indicating activated autophagy under 3D culture conditions. Interestingly, the elevated level of p62 persisted even in 3D–2D MSCs (Additional file 5: Fig.S5C). However, obtained results partially correlate with western blot data (Additional file 5: Fig. S5D and S5D’). Observed increased protein level of p62 in 16p 2D-MSCs and 3D-MSCs is opposite to decreased p62 level in early passage MSCs as well as 1p 3D-2D MSCs, thus suggests autophagy activation (Additional file 5: Fig. S5D and S5D’). To further investigate the role of autophagy activation in ability of senescent MSC to acquire early passage phenotype in 3D culture, we treated MSCs with chloroquine, known as autophagy inhibitor [37], before 3D-spheroid formation. Our results showed that inhibition of autophagy during 3D-spheroid formation led to delayed rejuvenation effects, as evidenced by high levels of SA-β-gal activity in treated 3D–2D MSCs compared to untreated cells **(**Additional file 5: Fig. S5E). These findings suggest that autophagy may play a critical role in ability of senescent MSCs acquire the phenotype of early passage MSCs.

### MSCs reduce their morphological heterogeneity upon 3D Cultivation

**Fig. 6:**
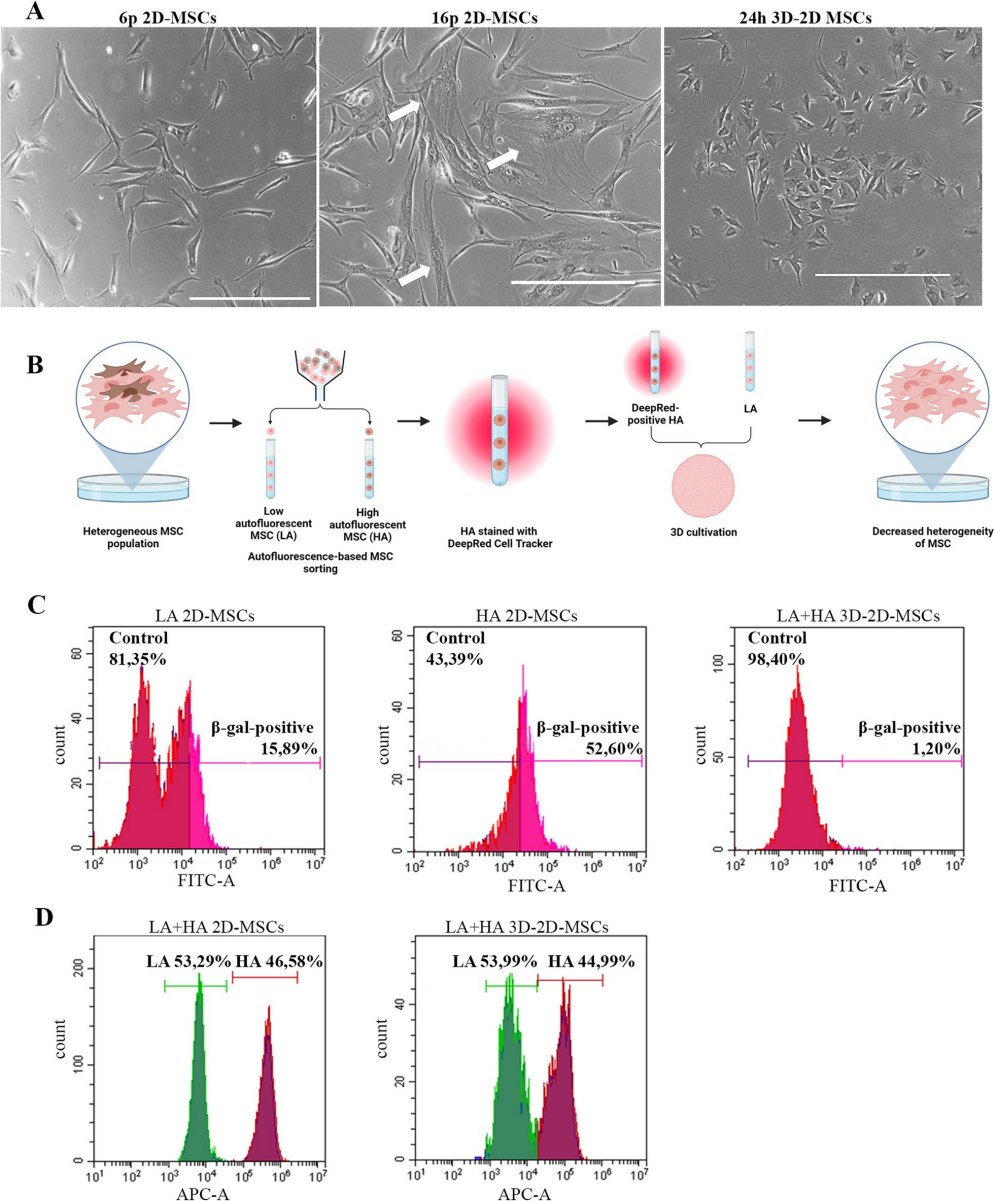
The assessment of selective elimination of senescent cells in 3D-MSCs. (**A**): Representative images of 2D-MSCs after 6 passages, 16 passages under 2D cell culture and after 1 passage under 3D-2D cell culture. White arrow points at senescent cell. Scale bar 400 μm. (**B**): a scheme of autofluorescence-based sorting experiment. (**C**): Flow cytometry analysis of (SA)-β-galactosidase activity of low autofluorescent (LA) 2D-MSCs, of high autofluorescent (HA) 2D-MSCs and of low and high autofluorescent (LA + HA) 3D-2D MSCs. (**D**): Flow cytometry analysis of distribution stained (HA) and unstained (LA) MSCs before 3D cell culturing and after

Human MSCs often exhibit heterogeneity in cell size and shape when cultured in vitro [3]*.* Heterogeneous hMSCs differ in the repertoire of expressed genes, secreted molecules and this may negatively affect the differentiation potential and immunomodulatory capabilities of cells, which may hamper the regenerative potential and widespread clinical application of MSCs [55]*.* In our study, we observed that after 16 passages, 2D-MSCs displayed a heterogeneous population consisting of spindle-like cells and a large proportion of abnormally enlarged cells with an increased ratio of the cytoplasm area to the nucleus in comparison to MSCs after 6 passages (Fig. 6A). However, 3D culturing followed by a 3D–2D transition led to a decrease in cell size and reduced heterogeneity in cell size (Fig. 6A). This is particularly interesting because abnormally enlarged cell size is considered to be another very important replicative senescence marker.

### The rejuvenation of MSCs in 3D spheroids is not due to the selective elimination of senescent cells

Having found a number of morphological and physiological changes that occur to MSCs in 3D culture and subsequent re-plating in 2D culture, the question arose: does 3D-culturing and 3D–2D transition facilitate a true senescence marker loss in every cell or rather does this technique contribute to the death of the senescent cells? Thus, cell sorting based on autofluorescence was used in order to distinguish between the two subpopulations: cells with low autofluorescence rate (which were as assumed to be the cells with normal size and morphology) and cells with high autofluorescence rate (which were assumed to be the enlarged MSCs) (Fig. 6B). Next, in these two subpopulations, SA-β-Gal activity was examined. We found that in 2D-MSCs after passage 16 only 16% of cells with low autofluorescence were SA-β-Gal positive, while more than 50% of cells with high autofluorescence exhibited elevated SA-β-Gal activity (Fig. 6C).

Then, to prove that the observed changes are not due to a negative selection of senescent cells in spheroids, the 2D-MSC subpopulation with high autofluorescence was labeled with a fluorescent dye Cell Tracker Deep Red and reunited with unlabeled low autofluorescent subpopulation in a ratio 1:1 to form 3D spheroids (Fig. 6D). Following three days in 3D spheroids and after 3D–2D transition the distribution of labeled and unlabeled MSCs and SA-β-Gal activity of them were assessed. According to obtained results, the ratio 1:1 retained as 45% of total cells were Deep Red-positive (Fig. 6D). Importantly, after 3D–2D transition no enlarged cells in vitro were observed and the number of SA-β-Gal-positive cells drastically declined to 1.2%. Given that, we suggest that a positive reversing effect on cellular senescence and heterogeneity occurs while cells are cultured in 3D, rather than an elimination of senescent cells (Fig. 6C).

## Discussion

The use of MSCs in regenerative medicine has gained significant attention due to their ability to secrete a myriad of trophic molecules and differentiate into various cell types of connective tissue. In order to yield a relevant cell number for research or clinical purposes, researches often use conventional 2D culturing due to its ease of use. However, this approach has its limitations. For instance, it cannot mimic the physiological microenvironment, thus obtained results cannot be fully considered as relevant and accurate. Therefore, 3D cell culture has emerged as more attractive alternative for expanding MSCs as it creates more physiological microenvironment [56]. Indeed, numerous studies reveal that under 3D culture conditions MSCs enhance their therapeutic potential which is crucial for clinical purposes [10, 24, 57].

These enhanced features include augmented osteogenic differentiation and migration ability thus wound healing potency, promotion of anti-inflammatory responses, and angiogenesis. A plethora of research prove it, but mostly in the context of paracrine activity of 3D-MSCs [58–60]. Upon 3D culture MSCs change drastically the secretome; both in vitro and in vivo experiments using 3D-MSCs conditioned medium (CM) show increased regenerative capacity of such medium [61, 62].

Our study demonstrates for the first time that upon 3D culturing and further spheroids dissociation and culturing in 2D, the resulting 3D–2D MSCs retain and even increase osteogenic differentiation. This suggests that the enhanced regenerative potential of these cells goes beyond their paracrine activity. Moreover, our data of vinculin and β-catenin expression analysis demonstrated that 2D-MSCs after prolonged 2D culture (passaged 16 times), had less pronounced adhesion plaques. This may indicate a more elaborate ECM deposition and a loss of firm adhesion with the substrate in the late-passage 2D-MSC cells. This reduction in the focal adhesion size associates with the diminished migration ability of these late-passage 2D-MSCs. This will affect the regenerative potential of these cells.

Conversely, 3D–2D MSCs demonstrate enhanced wound healing potency and highly migrating phenotype with strong focal adhesions and polarized leading edge. Thus, we suggest that 3D–2D MSCs may become a reliable and promising source for future regenerative medicine applications as replicative senescence is among major obstacles to the clinical application of MSCs [25]. Previous study has established the potency of 3D culture to delay replicative senescence effect, primarily via colony forming unit assay which reflects the clonogenicity of MSCs [24]. In this study, we sought to provide further evidence 3D culturing has an effect of reversing cellular senescence by examining various replicative senescence-associated markers. We analyzed cell cycle progression before and after 3D culturing and found a significant increase in the number of MSCs in S-phase. These results do not contradict with some previous studies that have suggested that MSCs within 3D spheroids do not proliferate [10] our findings suggest that 3D culturing with subsequent spheroid dissociation promotes cellular proliferation. Additionally, we examined the surface expression of CD146 in 3D–2D MSCs, as expression of this surface marker is highly dependent on the cellular and molecular microenvironment and declines in senescent cells [40, 44]. Our data demonstrates that long-term 2D cultivation is accompanied by CD146 expression decline, but the number of CD146-positive MSCs increase significantly upon 3D culturing. Furthermore, we observed a decline in SA-β-Gal activity, a common senescence-associated marker, and lysosomal activity upon 3D culturing.

Under 3D cell culture, MSCs undergo a significant morphological transformation, that is, transition from flat spindle-like cells to small, rounded cells [24]. This altered morphology persists even after spheroid dissociation and subsequent 3D-2D culturing.

To investigate this phenomenon, we focused on the actin cytoskeleton, which plays a crucial role in various cellular functions and undergoes drastic changes under 3D culture conditions. Due to the tension-dependent nature of the actin cytoskeleton, it exhibits significant differences between 2 and 3D cultured cells [63]. Specifically, the Young’s elastic modulus of conventional tissue culture plastic is in the gigapascal (GPa) range, while that of 3D spheroids is in the pascal (Pa) range [64, 65]. Our findings on actin filaments in 3D-MSCs are consistent with Zhou et al. observation of a loss of tension in actin filaments within 3D-MSCs [27]. Interestingly, Zhou et al. also reported that the loss of actin cytoskeleton tension is linked to upregulation of the pluripotency gene *NANOG* [27]. This is noteworthy given that several studies have shown upregulation of pluripotency genes within 3D-MSCs [27, 66]. While the upregulation of pluripotency genes in adult cells remains a topic of debate, dedifferentiation of MSCs is a promising approach to overcome replicative senescence [9].

Actin and actin-associated proteins regulate gene expression as well as other cellular events [67, 68]. Actin cytoskeleton modulates organelles positioning, in particular it regulates the positioning of Golgi apparatus [31]. In our experiments we found a loss of common ribbon-like GA structure within 3D-MSCs, which correlates with condensed actin filaments. We propose that senescence may have triggered Golgi apparatus fragmentation which persists in 3D-MSCs.

GA serves as a hub for the assembly of numerous protein complexes, one of them is mTOR [33, 34]. It is widely accepted that mTOR is one of the main senescence governors [35] while using its inhibitors such as rapamycin show attenuation of senescent phenotype [69]. Given the disrupted structure of GA, we examined mTOR localization in 2D and 3D MSCs and discovered for the first time that mTOR has both cytoplasmic and nuclear localization within 3D-MSCs. The nuclear localization of mTOR has been observed in both budding yeast and mammalian cells [53, 70, 71]. It is known that nuclear mTOR (nmTOR) plays a critical role in the regulation of ribosomal genes expression as a transcriptional factor [71]. Additionally, nmTOR has been found to bind genes involved in canonical mTOR signaling, amplifying or suppressing mTOR signaling in a feedforward manner in response to physiological stimuli. Interestingly, mTOR is known as a negative regulator of autophagy, while unc-51-like autophagy-activating kinase 2 (ULK2) is a target of nmTOR [71], and autophagy was observed in MSCs in 3D culture [66]. Autophagy has an ambiguous role in senescence. Indeed, is has been shown that old bone marrow-derived MSCs demonstrated low autophagic activity rate in comparison with MSCs isolated from young mice [72]. Additionally, impaired autophagy can lead to an accumulation of reactive oxygen species (ROS), which triggers senescence by disrupting cellular homeostasis [73]. On the other hand, studies using cancer-associated fibroblasts have shown that overexpression of senescence-associated p16 and p21 can drive autophagy activation [74]. Therefore, it is crucial to gain a deeper understanding of the role of autophagy activation in cell behavior in 3D culture.

We suggest that mTOR sequestration in the nucleus is a regulatory mechanism of its activity in 3D-MSCs. We revealed that upon 3D culturing phospho-mTOR (Ser2448) expression declines drastically; it is known that phospho-mTOR represents an active form of the protein [53]. Together these findings suggest that the localization and activity of mTOR may be regulated by cellular context, such as 2D vs. 3D culturing, and this regulation may play a role in senescence and cellular size regulation.

Notably, senescent MSCs with abnormally enlarged morphology are not observed upon 3D culturing conditions. This led us to investigate whether 3D culturing could facilitate the death of true senescent MSCs, given the harsh microenvironment within spheroids characterized by low nutrients and high hypoxia [10]. By utilizing cell sorting [42] and staining for senescent cells, we combined these cells with MSCs exhibiting normal morphology and subjected them to 3D culturing. Our findings demonstrate, for the first time that senescent cells acquire phenotype of early passage cells in 3D culture.

Despite the potential, there are limitations such as lack of insights into the exact mechanisms underlying the ability of senescent MSCs to acquire the phenotype of early passage MSCs. It is also uncertain if the effect reversing senescence can be sustained during long-term cultivation. Additionally, it would be beneficial to broaden the range of MSCs investigated, derived from diverse sources, to further elucidate the patterns of processes that occur in 3D cultures.

The results of this study carry significant implications for the advancement of innovative approaches aimed at maximizing the therapeutic capabilities of MSCs in the field of regenerative medicine.

## Conclusion

We have demonstrated an ability of senescent MSCs acquire phenotype of early passage MSCs through 3D-culture and 3D–2D transition. Our findings also reveal that the formation of 3D-spheroids triggers significant rearrangement of F-actin and changes in vinculin and β-catenin expression. Notably, upon the 3D culture of MSCs regular GA structure was revealed to be lost, and the positive senescence regulator mTOR was found to be localized in the nucleus. One potential future outcome of this study is gaining a deeper understanding of how cytoskeleton rearrangement and autophagy activation contribute to reversal senescence effect. Additionally, a comprehensive analysis of the secretome and transcriptome of MSCs in 3D culture could provide valuable insights into the critical rejuvenation processes. By exploring these avenues, we can enhance our understanding of the mechanisms underlying rejuvenation. These findings have important implications for the development of novel strategies for enhancing the therapeutic potential of MSCs in regenerative medicine.

### Supplementary Information

**Additional file 1**. **Figure S1**. Analysis of cell cycle and surface markers expression. (**A**): cell cycle analysis of 7p 3D-2D MSCs; (**B**): expression of CDKN1A and CDKN2A in 2D-MSCs (6p and 16p), 3D-MSCs (24h, 48h and 72h) and 3D-2D MSCs (1p). Data are shown as mean ± SD, n = 3, with significance difference indicated with asterisks (**** - p < 0.0001). (**C**): expression of CD146 in 3D-MSCs. (**D**): expression of CD90 in 2D-MSCs (4p and 16p), 3D-MSCs and 3D-2D MSCs (1p).

**Additional file 2**.**Figure S2**. (**A**): Adipogenic differentiation of 2D-MSCs and 3D-2D MSCs (Left panel: control, right panel: adipogenic differentiation). Scale bar 100 µm. (**B**): Chondrogenic differentiation of 2D-MSCs and 3D–2D MSCs. (Left panel: control, right panel: chondrogenic differentiation). Scale bar 400 µm. (**C**): Wound healing potency curve of 16p 2D-MSCs and 1p 3D-2D MSCs. Time point 0,5 hour.

**Additional file 3**. **Figure S3**. Analysis of cytoskeletal and adhesion proteins and their coding genes expression in MSCs in various culture conditions. (**A**): ACTB, VCL and CTNNB1 expression in 2D-MSCs (6p and 16p), 3D-MSCs (24h, 48h and 72h) and 3D-2D MSCs (1p). Data are shown as mean ± SD, n = 3, with significance difference indicated with asterisks (ns – not significant, ** - p < 0.01, *** - p < 0.001, **** - p < 0.0001). (**B**): Representative Western blot analysis of F-actin, Vinculin and β-catenin in 2D-MSCs (6p and 16p), 3D-MSCs (48h) and 3D-2D MSCs (1p). Data are shown as mean ± SD, n = 3, with significance difference indicated with asterisks (ns – not significant, ** - p < 0.01, *** - p < 0.001, **** - p < 0.0001). Full-length blots are represented in Additional file 2: Supplementary Figure 1.

**Additional file 4**. **Figure S4**. Immunofluorescent analysis of p230 and phospho-mTOR (Ser2448) proteins in 2D-MSCs (6p and 16p) and 3D-2D MSCs (1p). Scale bar 50 µm. Red Asterisk points MSCs with early passage phenotype where p230 and phospho-mTOR (Ser2448) colocalize. White Asterisk points at senescent MSCs with dispersed GA (p230 distribution pattern). Abbreviations: DAPI – 4′,6-diamidino-2-phenylindole.

**Additional file 5**. **Figure S5**. (**A**): Analysis of TFEB, ATG5, ATG16L1, MAP1LC3B, GARABAPL expression in 2D-MSCs (6p and 16p), 3D-MSCs (24h, 48h and 72h) and 3D-2D MSCs (1p). Data are shown as mean ± SD, n = 3, with significance difference indicated with asterisks (* - p < 0.05, ** - p < 0.01, *** - p < 0.001, **** - p < 0.0001); (**B**): Electron microscopy observation of autophagosomes in 3D-MSCs, Red Asterisk point at double-layer membrane structures – autophagosomes. Scale bar 1 µm; (**C**): Immunofluorescent analysis of p62 staining pattern in 2D-MSCs (6p and 16p), 3D-MSCs and 3D-2D MSCs (1p). Scale bar 50 µm; (**D**, D’): Representative Western blot analysis of p62 protein level in 2D-MSCs (6p and 16p), 3D-MSCs (48h) and 3D-2D MSCs (1p). Data are shown as mean ± SD, n = 3, with significance difference indicated with asterisks (ns – not significant, ** - p < 0.01, **** - p < 0.0001). Full-length blots are represented in Additional file 2: Supplementary Figure 1; (**E**): Analysis of SA-β-gal activity in 3D-2D MSCs. Left panel: control. Right panel: chloroquine treated (5 µM). Scale bar 400 µm.

**Additional file 6**.**Additional file 2**: Supplementary Figure 1. Uncropped full-length blots. (A-D): correspond to Supplement Figure S3B. 1 stands for 2D-MSCs 6p, 2 – 2D-MSCs 16p, 3 – 3D-MSCs, 4 – 3D-2D MSCs 1p; (E-H): correspond to Figure 5C. “-“ and “+” stand for chloroquine non-treated (-) and treated (+). Chloroquine-treated samples are not discussed in the manuscript. Bands shown on figure 5C are presented in black boxes. 1 stands for 2D-MSCs 6p, 2 – 2D-MSCs 16p, 3 – 3D-2D MSCs 1p, 4 – 3D-MSCs 24h, 5 – 3D-MSCs 48h; (I, J): correspond to Supplement Figure S5D. 1 stands for 2D-MSCs 6p, 2 – 2D-MSCs 16p, 3 – 3D-MSCs, 4 – 3D-2D MSCs 1p.

## Data Availability

Not applicable.

## Abbreviations

2D: Two-dimensional
3D: Three-dimensional
DAPI: 4′,6-Diamidino-2-phenylindole
ECM: Extracellular matrix
GA: Golgi apparatus
GAPDH: Glyceraldehyde-3-phosphate dehydrogenase
MSCs: Mesenchymal stem cells
mTOR: Mammalian target of rapamycin
mTORC1: MTOR Complex 1
nmTOR: Nuclear mTOR
SA-β-Gal: Senescence-associated beta-galactosidase
