# Supplementary figures and images for: Mesenchymal stem cells lose the senescent phenotype under 3D cultivation
Source: Stem Cell Res Ther. 2023 Dec 18;14:373. doi: 10.1186/s13287-023-03599-8 (PMC10729581; doi:10.1186/s13287-023-03599-8)

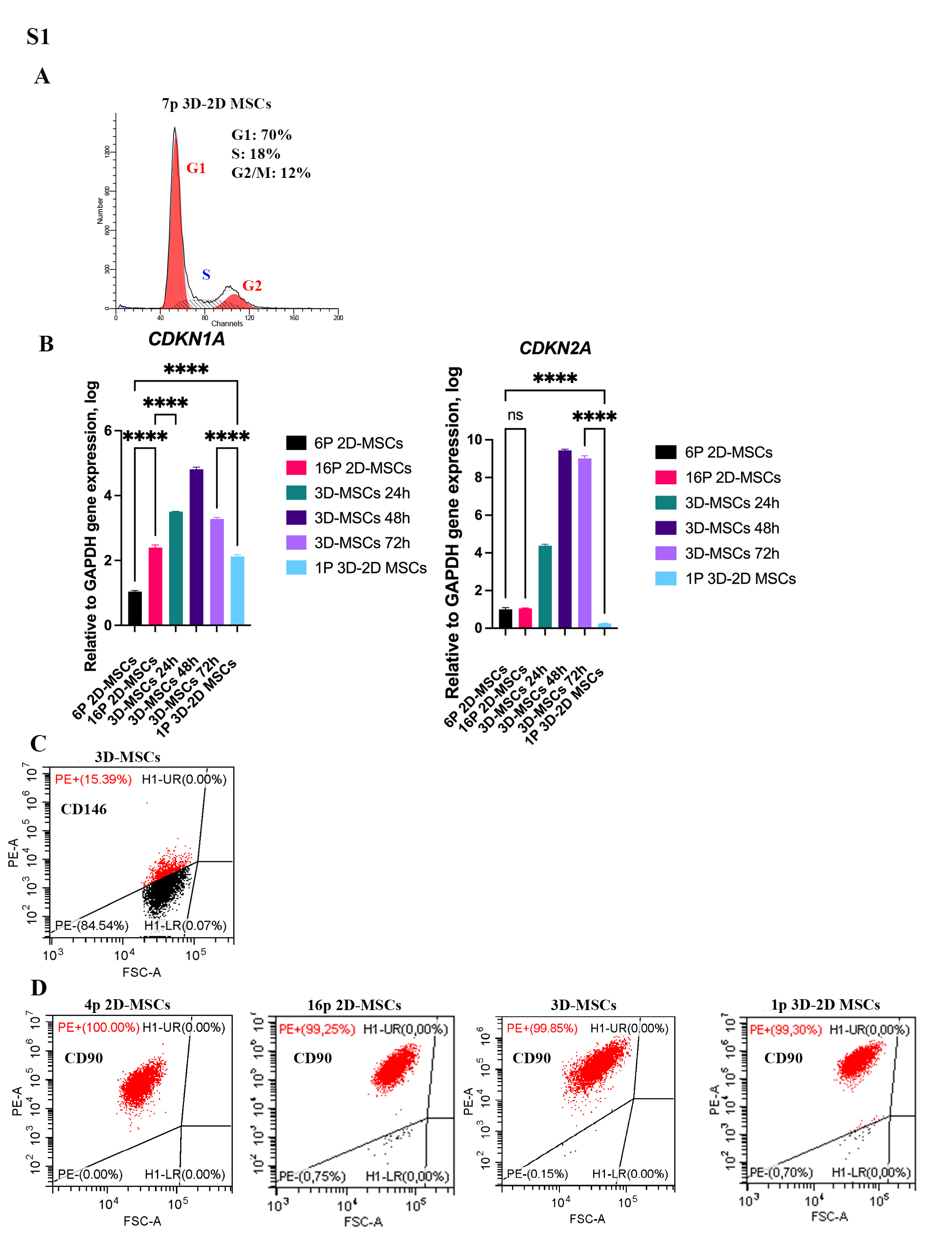

Supplement: Supplementary file 1 — Additional file 1. Figure S1. Analysis of cell cycle and surface markers expression. (A): cell cycle analysis of 7p 3D-2D MSCs; (B): expression of CDKN1A and CDKN2A in 2D-MSCs (6p and 16p), 3D-MSCs (24h, 48h and 72h) and 3D-2D MSCs (1p). Data are shown as mean ± SD, n = 3, with significance difference indicated with asterisks (**** - p < 0.0001). (C): expression of CD146 in 3D-MSCs. (D): expression of CD90 in 2D-MSCs (4p and 16p), 3D-MSCs and 3D-2D MSCs (1p). [file 13287_2023_3599_MOESM1_ESM.tif]

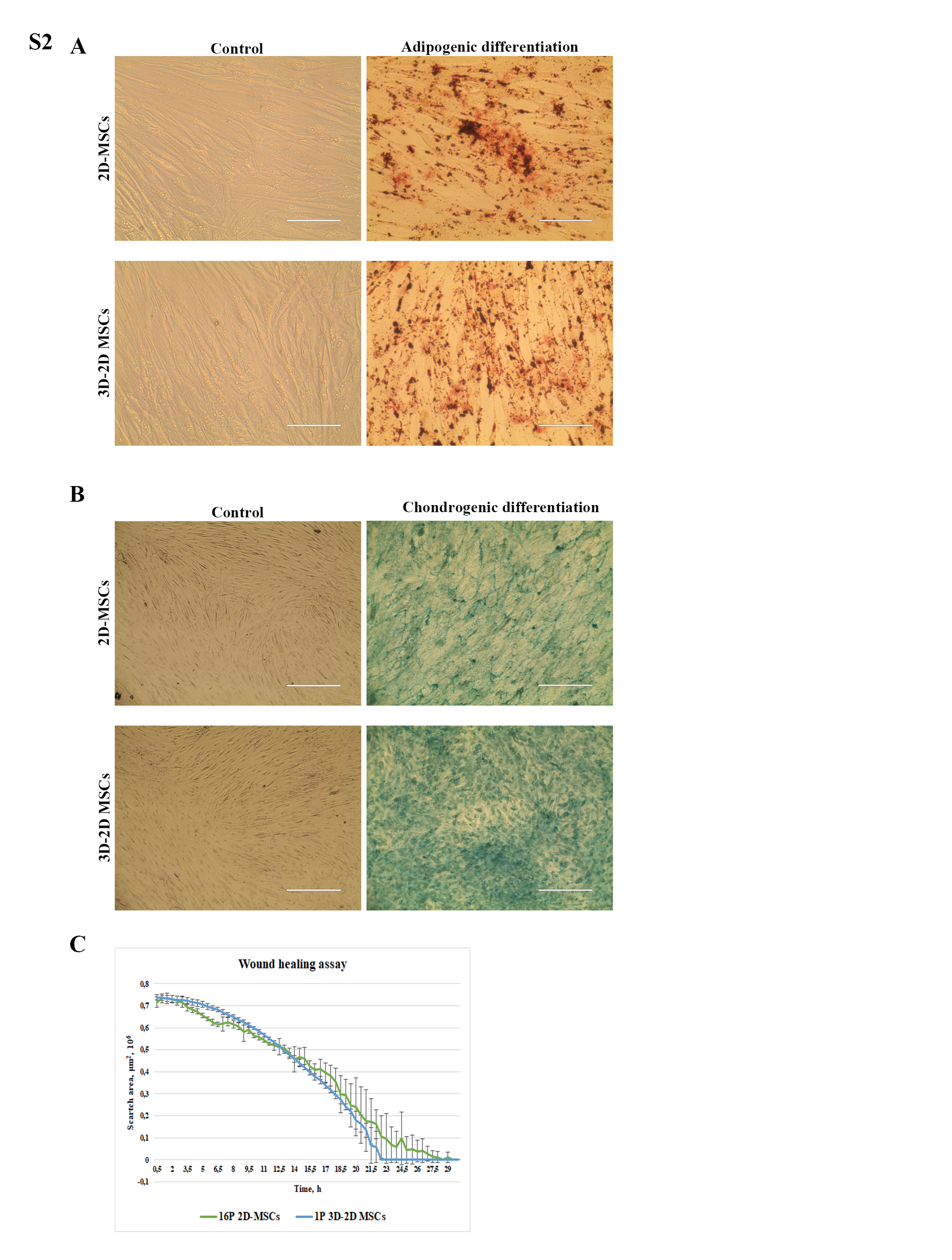

Supplement: Supplementary file 2 — Additional file 2.Figure S2. (A): Adipogenic differentiation of 2D-MSCs and 3D-2D MSCs (Left panel: control, right panel: adipogenic differentiation). Scale bar 100 µm. (B): Chondrogenic differentiation of 2D-MSCs and 3D–2D MSCs. (Left panel: control, right panel: chondrogenic differentiation). Scale bar 400 µm. (C): Wound healing potency curve of 16p 2D-MSCs and 1p 3D-2D MSCs. Time point 0,5 hour. [file 13287_2023_3599_MOESM2_ESM.tif]

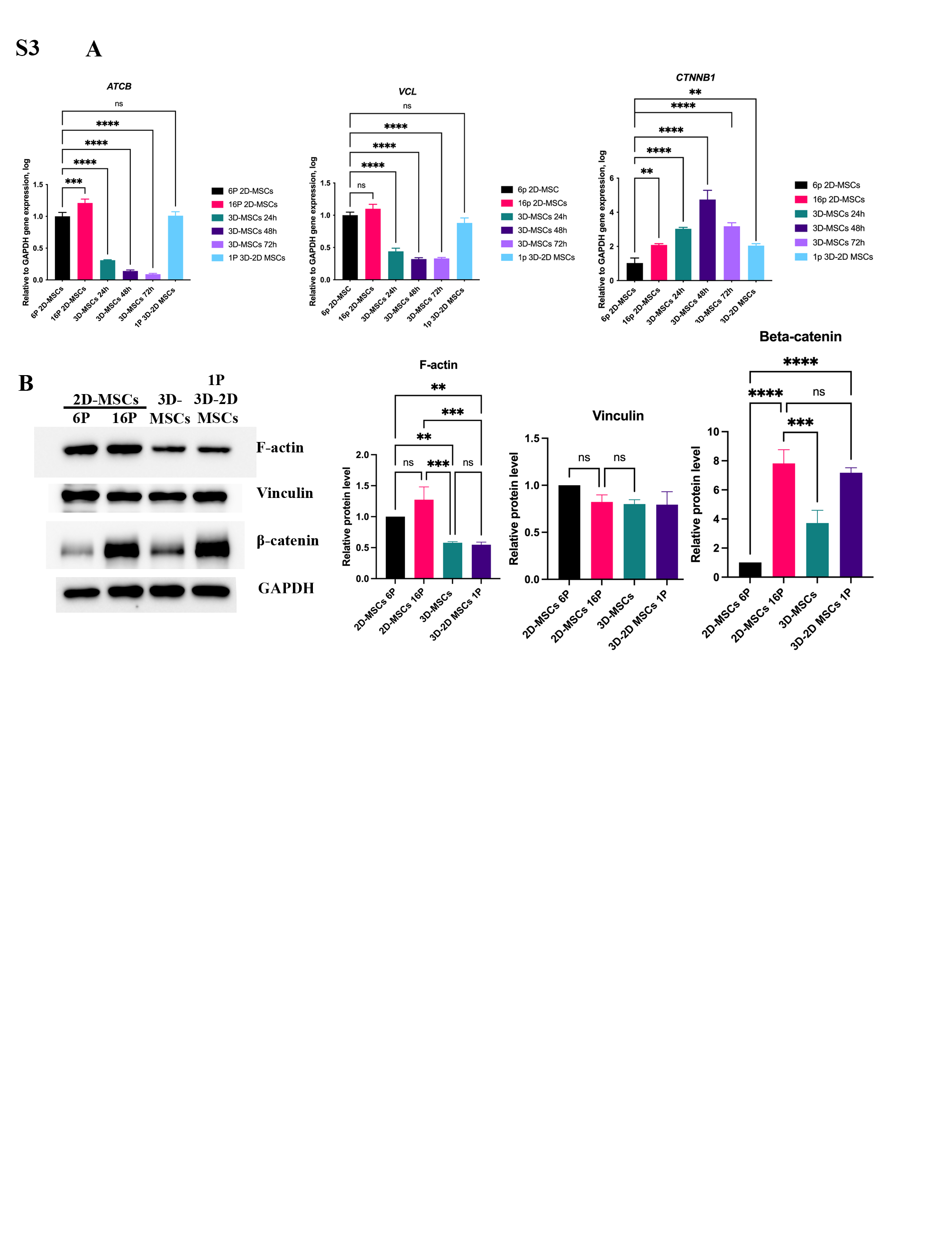

Supplement: Supplementary file 3 — Additional file 3. Figure S3. Analysis of cytoskeletal and adhesion proteins and their coding genes expression in MSCs in various culture conditions. (A): ACTB, VCL and CTNNB1 expression in 2D-MSCs (6p and 16p), 3D-MSCs (24h, 48h and 72h) and 3D-2D MSCs (1p). Data are shown as mean ± SD, n = 3, with significance difference indicated with asterisks (ns – not significant, ** - p < 0.01, *** - p < 0.001, **** - p < 0.0001). (B): Representative Western blot analysis of F-actin, Vinculin and β-catenin in 2D-MSCs (6p and 16p), 3D-MSCs (48h) and 3D-2D MSCs (1p). Data are shown as mean ± SD, n = 3, with significance difference indicated with asterisks (ns – not significant, ** - p < 0.01, *** - p < 0.001, **** - p < 0.0001). Full-length blots are represented in Additional file 2: Supplementary Figure 1. [file 13287_2023_3599_MOESM3_ESM.tif]

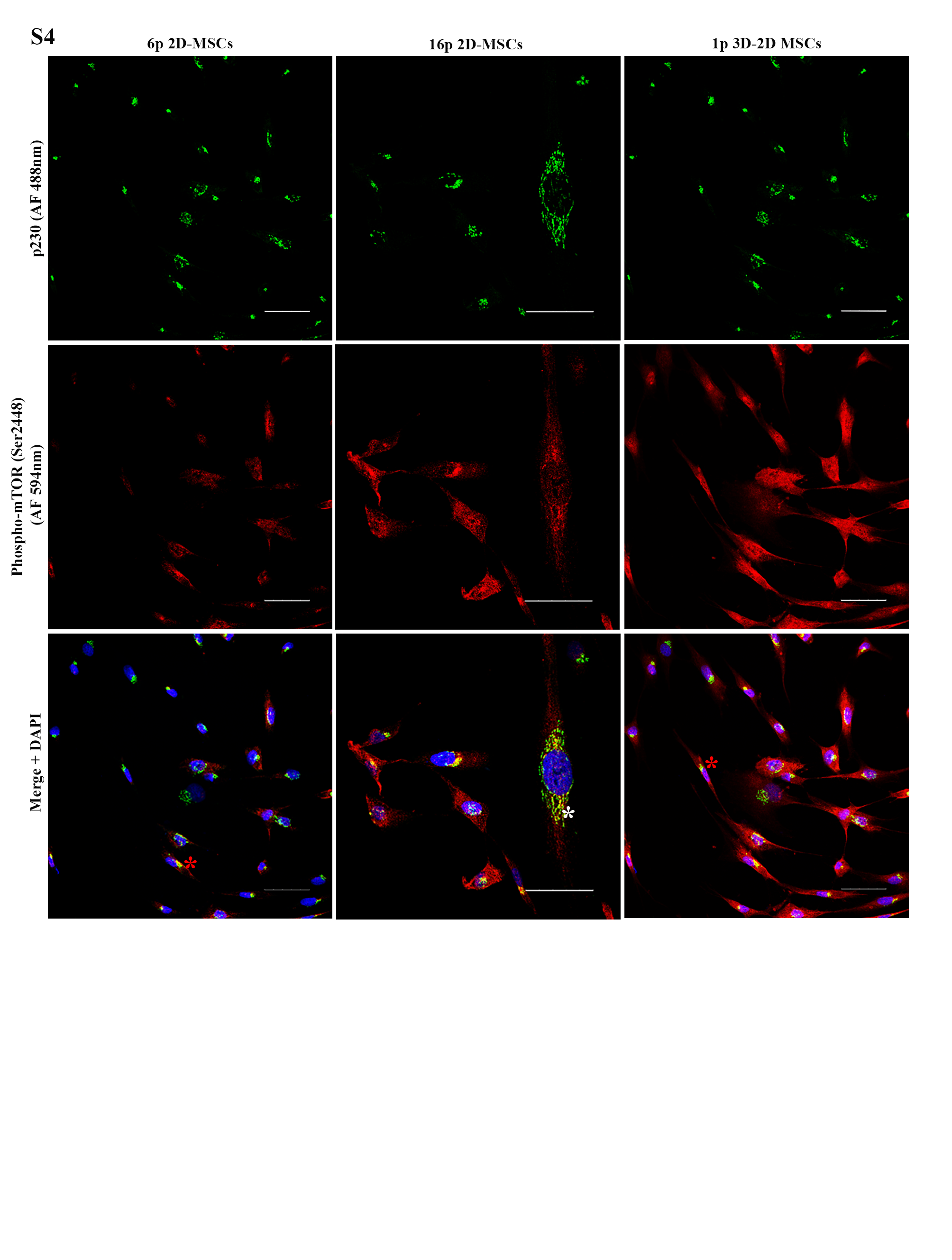

Supplement: Supplementary file 4 — Additional file 4. Figure S4. Immunofluorescent analysis of p230 and phospho-mTOR (Ser2448) proteins in 2D-MSCs (6p and 16p) and 3D-2D MSCs (1p). Scale bar 50 µm. Red Asterisk points MSCs with early passage phenotype where p230 and phospho-mTOR (Ser2448) colocalize. White Asterisk points at senescent MSCs with dispersed GA (p230 distribution pattern). Abbreviations: DAPI – 4′,6-diamidino-2-phenylindole. [file 13287_2023_3599_MOESM4_ESM.tif]

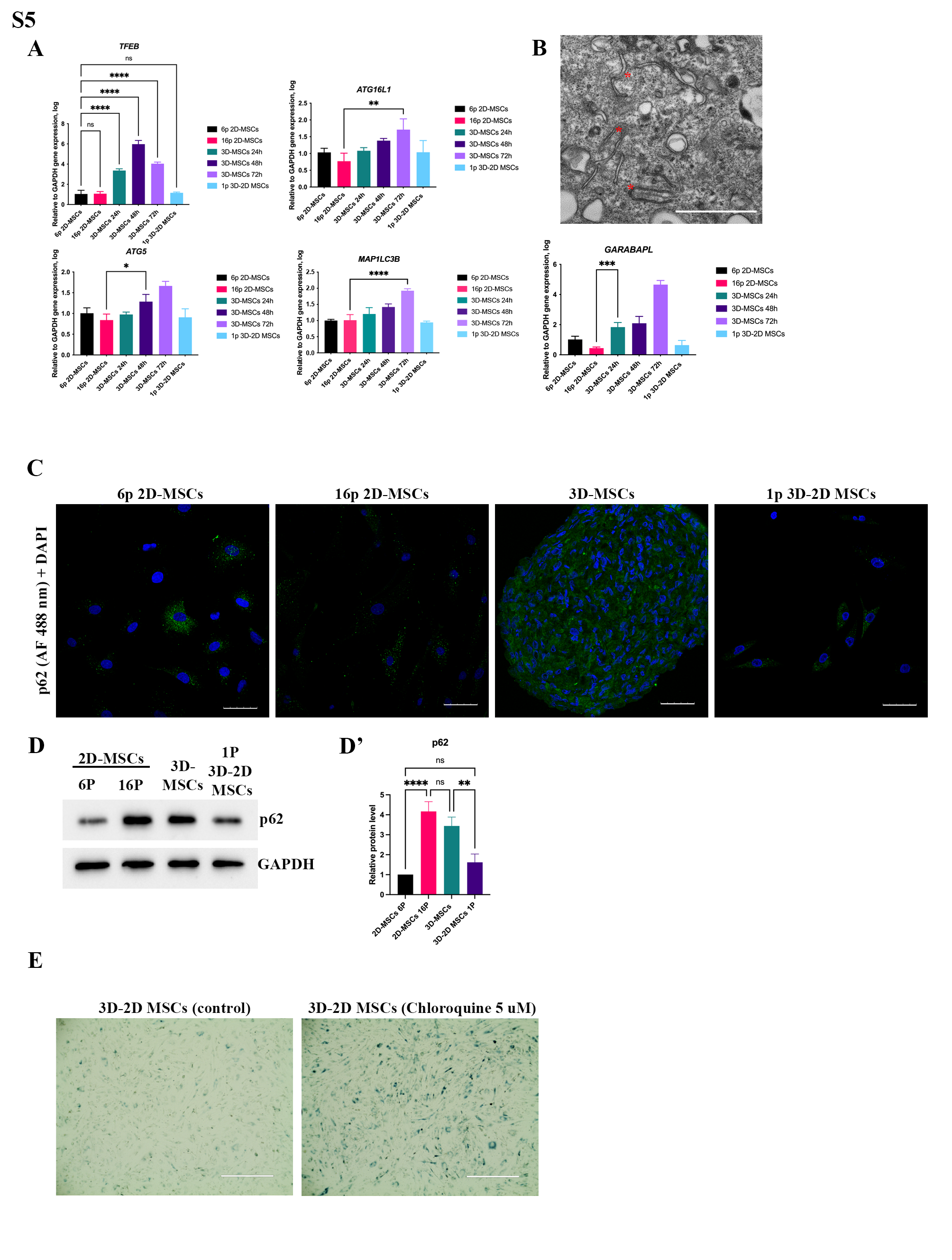

Supplement: Supplementary file 5 — Additional file 5. Figure S5. (A): Analysis of TFEB, ATG5, ATG16L1, MAP1LC3B, GARABAPL expression in 2D-MSCs (6p and 16p), 3D-MSCs (24h, 48h and 72h) and 3D-2D MSCs (1p). Data are shown as mean ± SD, n = 3, with significance difference indicated with asterisks (* - p < 0.05, ** - p < 0.01, *** - p < 0.001, **** - p < 0.0001); (B): Electron microscopy observation of autophagosomes in 3D-MSCs, Red Asterisk point at double-layer membrane structures – autophagosomes. Scale bar 1 µm; (C): Immunofluorescent analysis of p62 staining pattern in 2D-MSCs (6p and 16p), 3D-MSCs and 3D-2D MSCs (1p). Scale bar 50 µm; (D, D’): Representative Western blot analysis of p62 protein level in 2D-MSCs (6p and 16p), 3D-MSCs (48h) and 3D-2D MSCs (1p). Data are shown as mean ± SD, n = 3, with significance difference indicated with asterisks (ns – not significant, ** - p < 0.01, **** - p < 0.0001). Full-length blots are represented in Additional file 2: Supplementary Figure 1; (E): Analysis of SA-β-gal activity in 3D-2D MSCs. Left panel: control. Right panel: chloroquine treated (5 µM). Scale bar 400 µm. [file 13287_2023_3599_MOESM5_ESM.tif]

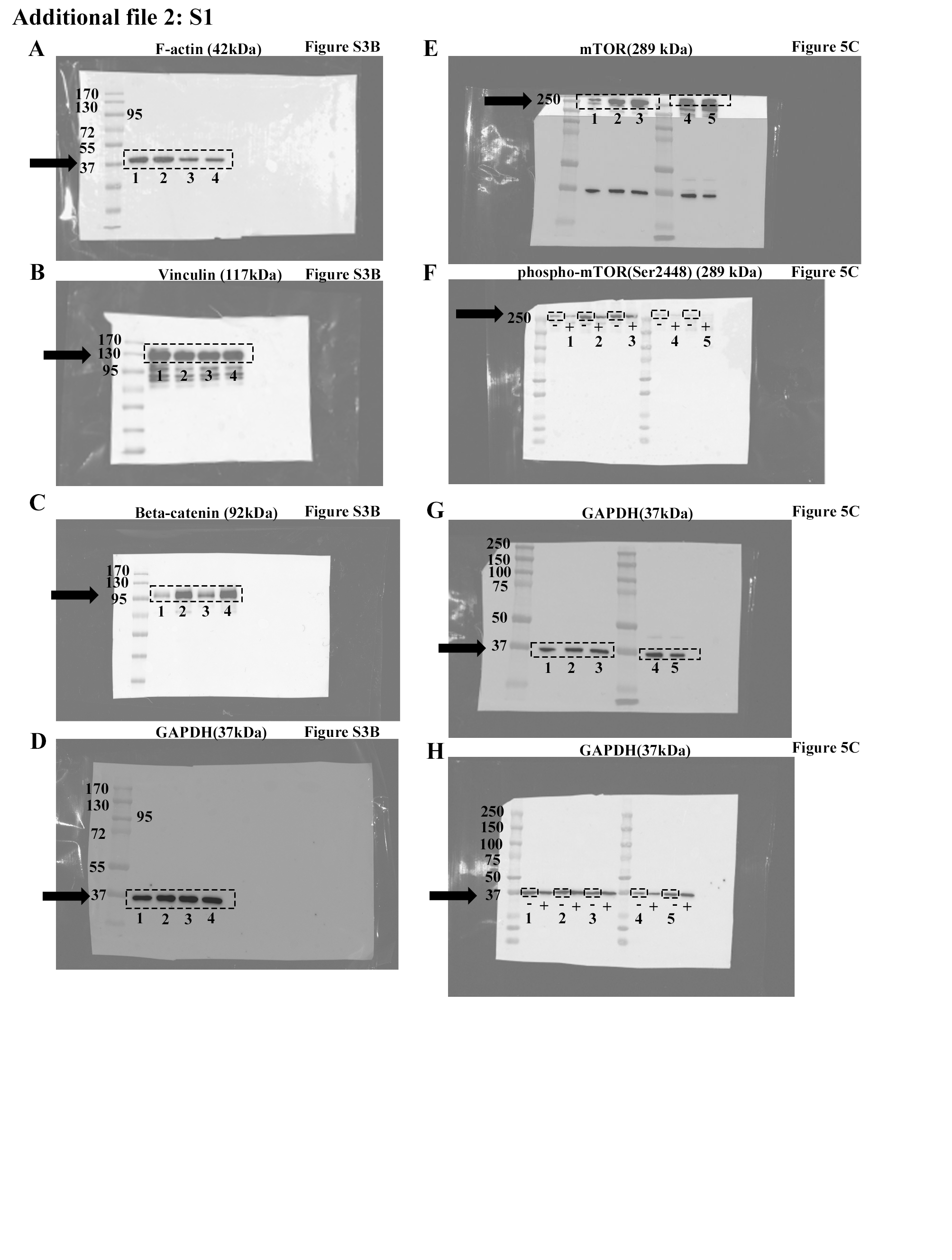

Supplement: Supplementary file 6 — Additional file 6.Additional file 2: Supplementary Figure 1. Uncropped full-length blots. (A-D): correspond to Supplement Figure S3B. 1 stands for 2D-MSCs 6p, 2 – 2D-MSCs 16p, 3 – 3D-MSCs, 4 – 3D-2D MSCs 1p; (E-H): correspond to Figure 5C. “-“ and “+” stand for chloroquine non-treated (-) and treated (+). Chloroquine-treated samples are not discussed in the manuscript. Bands shown on figure 5C are presented in black boxes. 1 stands for 2D-MSCs 6p, 2 – 2D-MSCs 16p, 3 – 3D-2D MSCs 1p, 4 – 3D-MSCs 24h, 5 – 3D-MSCs 48h; (I, J): correspond to Supplement Figure S5D. 1 stands for 2D-MSCs 6p, 2 – 2D-MSCs 16p, 3 – 3D-MSCs, 4 – 3D-2D MSCs 1p. [file 13287_2023_3599_MOESM6_ESM.tif]
